# The Pharmacological Mechanism of *Guchangzhixie* Capsule Against Experimental Colitis

**DOI:** 10.3389/fphar.2021.762603

**Published:** 2021-11-18

**Authors:** Jing Yan, Wei Yu, Chang Lu, Chen Liu, Guoliang Wang, Lu Jiang, Zizheng Jiang, Zheng Qin

**Affiliations:** ^1^ Department of Physiology, Jining Medical University, Jining, China; ^2^ Shandong University, Jinan, China

**Keywords:** inflammatory bowel disease, ulcerative colitis, macrophages, intestinal organoids, guchangzhixie capsule

## Abstract

Ulcerative colitis (UC) is the major type of inflammatory bowel disease (IBD) characterized by an overactive immune response and destruction of colorectal epithelium with intricate pathological factors. *Guchangzhixie* (GCZX) capsule, included in the Chinese Pharmacopoeia 2020, has been widely utilized against UC. However, the underlying molecular mechanisms have not been elucidated. In the present study, a murine model of experimental colitis was established by orally feeding 4% dextran sodium sulfate (DSS) for 5 days and subsequently subjecting to GCZX treatment for another 15 days. Network pharmacology analysis was performed to predict the pertinent mechanisms of GCZX capsule. Cellular experiments examining the functional changes of intestinal organoids (IOs), macrophages (Mφs), and human colon epithelial cell cells (NCM460 cell line) after GCZX therapy were performed. Sequencing of 16S rRNA was conducted on the stools from the mouse model. Liquid chromatography-mass spectrometry (LC–MS) was utilized to detect serum metabolites. As a result, DSS induced experimental colitis, and this induction was alleviated by GCZX treatment, as evidenced by rescued pathological symptoms in UC mouse models, such as rectal bleeding stopping, decreased levels of albumin, interleukin-17, as well as chemokine (C-X-C motif) ligand 1 (CXCL1), and reduction in colon length. Network pharmacology analysis showed that GCZX-target genes were enriched in pathogen-induced infections, inflammatory pathways, as well as neoplastic processes. DSS treatment decreased microbial diversity and led to the accumulation of pathological bacterial, which was reversed by GCZX capsule. PICRUSt2 (Phylogenetic Investigation of Communities by Reconstruction of Unobserved States) based on profiles of microbiota composition demonstrated a decreased incidence of infectious disease and cancers after GCZX therapy. In full accordance with these data, GCZX administration suppressed Mφ transition to pro-inflammatory phenotype, alleviated tumor necrosis factor-α (TNFα)-compromised IOs functions, and decreased the recruitment of Mφs by epithelial cells. We conclude that GCZX capsule is an effective drug for UC and its pharmacological mechanisms involve re-establishing an anti-inflammatory milieu and favoring mucosal healing.

## 1 Introduction

The relapsing and oncogenic nature of inflammatory bowel disease (IBD) dramatically affects patients’ quality of life, and searching for effective therapeutic strategies aiming at long-lasting clinical remission without serious adverse events has been the main topic in this field (Borren et al., 2021; van der Giessen et al., 2020; Voskuil et al., 2021). IBD is divided into Crohn’s disease (CD) and Ulcerative colitis (UC), sharing similar symptoms such as diarrhea, rectal bleeding, abdominal pain, and weight loss, and occurring in both adolescents and adults. Despite the similarity between these symptoms of CD and UC, there are some differences between these two, including affected sites and risk of cancer.

Most obviously, CD occurs in mouth, anus, as well as the entire intestine, while UC is often limited to colon and rectum (Veauthier and Hornecker, 2018). Moreover, UC patients bear a higher risk of developing cancer than patients with CD (Jess et al., 2006; Xue et al., 2018). However, both are pathologically characterized by epithelium disruption under a sustained pro-inflammatory microenvironment induced by a diverse range of factors, for instance, genetic susceptibility, physiological environmental dimension, psychological condition, and gut microbiota composition (Voskuil et al., 2021). Given the complexity of these factors, multi-target drugs show their superiority to combat IBD compared with exquisitely selective compounds.

*Guchangzhixie* (GCZX) capsule is an established drug included in the Chinese Pharmacopoeia 2020, which has been upgraded from Wumei (*Mume fructus*) pellet from Shang Han Lun (the oldest Chinese monograph on Cold Damage Diseases). GCZX capsule encompasses *Mume fructus* (***Mf***), *Zingiberis rhizoma* (***Zr***), *Aucklandiae radix* (***Ar***), *Corydalis rhizome* (***CRr***), *Coptidis rhizoma* (***CPr***), and *Papaveris pericarpium* (***Pp***). In this formula, ***Mf*** and ***Pp*** are astringent medicinal herbs that efficiently stop hemorrhoids and diarrhea. Additionally, ***Mf*** as the predominant component exerts anti-inflammatory as well as antibacterial effects (Choi et al., 2007; Chen et al., 2011; Kim et al., 2016; Xing et al., 2018). **Pp** has been used to treat chronic cough and cramp and alleviate human suffering (Cao et al., 2007). ***CRr*** and ***CPr*** share many common components that suppress inflammation (Kubo et al., 1994; Wang and Ng, 2001) and neoplasias (Peng et al., 2006; Chen et al., 2016; Wan et al., 2019), and represent strong antiviral (Wang and Ng, 2001) and antibacterial activity (Tian et al., 2020). In addition to its anti-inflammatory role (Endo et al., 2017; Endo et al., 2018; Tian et al., 2020), **Zr** has been utilized in combination with other herbs to reduce toxicity and optimize clinical efficacy (Peng et al., 2013). **Ar** alleviates diarrhea and gastric ulcer injury through modulating gastric emptying and intestinal propulsion (Huang et al., 2021), and numerous studies reported its antibacterial (Lee and Kim, 2020), anti-inflammatory (Wang et al., 2020a), and anti-tumor (Roy and Manikkam, 2015) roles, and regulation of bacterial composition (Hasson et al., 2013; Huang et al., 2021; Rocha et al., 2021). Logically, this formula combinates herbs that exert anti-inflammatory, antibacterial, and antidiarrheal effects. Nevertheless, the cellular mechanism of GCZX capsule lacks solid experimental validation.

In the present study, we ascertained the intervention effect of GCZX capsule on experimental colitis mice, discussed its mechanical mechanisms utilizing network pharmacology, and sequentially validated its therapeutic role by cellular experiments integrated with 16S rRNA sequencing.

## 2 Materials and Methods

### 2.1 Ethics Statement

All procedures and assays were approved by the Institutional Animal Care and Use Committee of Jining Medical University.

### 2.2 Establishing Component-Target Network of GCZX Components

Using Traditional Chinese Medicine Systems Pharmacology database and Analysis Platform (TCMSP) (Ru et al., 2014), the active components were obtained according to the suggested parameter information. The cutoff was greater or equal to 0.18 (Drug likeness, DL) and 20% (Oral bioavailability, OB). The targets of each active component were transformed into gene symbols of *Homo sapiens* species by the UniProt knowledge database (www.uniport.org).

### 2.3 Collecting Colitis-Related Genes

We collected colitis-related genes from five sources with the keyword “ulcerative colitis,” including GeneCards (Rebhan et al., 1997; Safran et al., 2010), DrugBank (Wishart et al., 2018), Online Mendelian Inheritance in Man (OMIM) (Hamosh et al., 2002), PharmGkb (Whirl-Carrillo et al., 2012), and Statistics of Therapeutic Target database (TTD) (Wang et al., 2020b) (Supplementary Table S1).

### 2.4 Herb–Ingredient–Target (HIT) Interaction Network

The shared genes between GCZX capsule and colitis were selected to build the HIT interaction network utilizing Cytoscape software (Su et al., 2014).

### 2.5 Protein-Protein Interaction (PPI) Network and Hub-Genes Calculation

Utilizing the STRING database (Search Tool for Retrieval of Interacting Genes/Proteins) (http://string-db.org/) (Szklarczyk et al., 2019), a PPI network based on the shared genes was computed with a confidence score ≥0.7 for significance and without disconnected nodes. Network nodes refer to proteins; edges do not mean the physically binding of two proteins, but the protein-protein interactions that they jointly facilitate a function. The thicker the edge is, the higher the confidence is.

Hub genes with a high-degree node were calculated by CytoNCA according to six indices, including closeness centrality, betweenness centrality, degree centrality, eigenvector centrality, local average connectivity, and network centrality (Tang et al., 2015). Genes above the median value of each index were selected per calculation. These indices were calculated three times to establish the final sub-network.

### 2.6 GO and KEGG Pathway Enrichment

Gene Ontology (GO) divides genetic functions into cellular component (CC), molecular function (MF), and biological process (BP) (Ashburner et al., 2000). Kyoto Encyclopedia of Genes and Genomes (KEGG) is a reference knowledge base for systematic interpretation of genes functions (Kanehisa and Goto, 2000). GO and KEGG enrichment analysis was executed by Bioconductor (R). Adjusted *p*-value below 0.05 was considered significantly enriched genes.

### 2.7 Experimental Validation

#### 2.7.1 Ulcerative Colitis Mouse Model and GCZX Treatment

***Mf***, ***Zr***, ***Ar***, ***CRr***, ***CPr***, ***Pp*** were mixed with the ratio of 4:1.3:1:1.3:1:1, added to 1,000 ml (1: 10 g/v) pure water, and boiled for 1 h.

A total of 45 C57BL/6 male mice (Cyagen, China) weighing 20–25 g were randomly divided into three groups (15 mice per group): control group, DSS group, and GCZX-treated DSS group. Mice were fed with 4% dextran sodium sulfate (DSS, MP Biomedicals) diluted in water for 4 days. Upon removal on day 5, GCZX solution was administered for another 2 weeks.

The disease activity index (DAI) score included measurements of stool, weight loss, fecal occult, and histology and was calculated as follows (Travis et al., 2013): (1) stool (normal = 0; soft = 1; very soft, semi-formed = 2; liquid, sticky, or unable to defecate = 3); (2) weight loss (no loss = 0; <5% = 1; 5–10% = 2; 10–20% = 3; >20% = 4); (3) bloody stool test (not positive within 2 min = 0; purple positive after 10 s = 1; light purple positive within 10 s = 2; heavy purple positive within 10 s = 3) (Leagene); and (4) indices of the histological scores included destruction of the epithelial monolayer, edema, crypt loss, and mucosa infiltration.

Tissues were fixed and embedded in paraffin for 72 h. Slice (3.5 μm thick) was stained with hematoxylin and eosin (H&E) and visualized on the microscope.

#### 2.7.2 GCZX Solution Preparation

To prepare GCZX solution for cellular experiments, we established a HIT interaction network with the hub genes and the active components of herbs. We selected the common components of these herbs based on TCMSP (Ru et al., 2014) and one typical ingredient with the highest degree in each herb acquired in the HIT interaction network: oleanolic acid (***Ar***, ***Mf***), beta-sitosterol (***Mf***, ***Zr***), kaempferol (***Mf***), quercetin (***Mf***, ***CPr*** and ***CRr***), morphine (***Pp***), palmatine, berberine and coptisine (***CPr*** and ***CRr***). The mixture of these compounds was diluted in DMSO (dimethyl sulfoxide) at a concentration of 10 ng/ml, and 20 ng/ml of each compound (All chemicals have purchased the chemicals in Yuanye Biology, China). The concentration of compounds representing each herb in the final solution was almost equal to the proportion in GCZX capsule. MTT (3-[4,5-dimethylthiazol-2-yl]-2,5 diphenyl tetrazolium bromide) assay was utilized to determine the toxicity in NCM460 cells (Human epithelial cell line) (Supplementary Figure S2), a concentration of 10 ng/ml was selected.

#### 2.7.3 Amplicon Sequencing Data Analysis

Raw data was primarily filtered by Trimmomatic (Bolger et al., 2014). Identification and removal of primer sequences were processed by Cutadapt (Kechin et al., 2017) and subsequent paired end reads were assembled by USEARCH and followed by chimera removal using UCHIME (Edgar et al., 2011). Original subreads were corrected to generate Circular Consensus Sequencing (CCS) reads by SMRT Link. CCS reads from different samples were distinguished based on barcode sequences. High-quality CCS reads were obtained after removing chimeras. Subsequently, OTU (operational taxonomic unit)/ASV (amplicon sequence variants) analysis was performed to cluster sequences with similarity over 97% and generating ASVs with conservative threshold for OTU filtration (0.005%). Species annotation and taxonomic analysis, diversity analysis including alpha and beta diversity, significant difference analysis, and functional prediction were performed (Supplementary method) (PRJNA757221).

#### 2.7.4 Identification of the Chemical Constituents of Serum From GCZX-DSS Mice

A Hybrid Quadrupole-Time-of-flight (TOF) Liquid Chromatography with tandem mass spectrometry (LC/MS/MS) was used. A total of 100 μl serum was added to 1 ml 80% methanol under ultrasonication for 15 min. After 1,200 r/min centrifugation for 10 min, the filtration was collected through a 0.45 μm membrane filter and injected into Hybrid TOF LC/MS/MS (Triple TOF 5600+, AB Sciex Instruments) to identify the chemical constituents followed the previous instructions (Dunn et al., 2011).

Chemical identification was performed on a connected system of LC-30 (Shimadzu)-Hybrid Quadruple time-of-flight mass spectrometer (TOF MS) with electrospray ionization source (ESI). InerSustain C18 column (Shimadzu, 100 × 2.1 mm, 2 µm) was used to perform chromatographic separation with a flow rate of 0.3 ml/min at 35°C. Mobile phase system was composed of Equate A (acetonitrile) and Equate B (0.1% HCOOH-H_2_O): 4 min (A:5%:B:95%), 8 min (A:20%:B:80%), 2 min (A:15%:B:75%), 2 min (A:46%:B:54%), 3 min (A:100%:B:0%), 1 min (A:5%:B:95%).

Following are the instrumental settings: both ion source gas 1 and gas 2 were 50 psi, curtain gas (CUR) was 25 psi, source temperature was 500°C in positive mode while 450°C in negative mode, ion spray voltage floating (ISVF) was 5500 V in positive mode while 4400 V in negative mode, TOF MS scan range was 100–1200 Da, product ion scan range was 50–1000 Da, TOF MS scan and product ion scan accumulation time was 0.2 and 0.01 s, respectively. Data was acquired in information-dependent acquisition (IDA) with high sensitivity mode, collision energy was 35 ± 15 eV, and declustering potential was ±60 V (Supplementary Table S1) (Supplementary Figure S1).

#### 2.7.5 Macrophage Isolation and Phagocytosis Experiment

Peritoneal macrophage (Mφ) isolation: Mice were sacrificed and the peritoneal liquid was collected. The pallet was diluted with RPMI1640 medium (Thermo Fisher, United States).

Bone marrow-derived Mφ (BMDM) culturing: Femurs from 4 weeks-age mice were collected and bone marrow was flushed out with cold PBS. Blood cell lysis buffer was added to the pellet for 5 min and the acquired cell medium was filtered by a 10 μm cell filter. After centrifugation, cells were dilated with complete solution (RPMI1640, 10% fetal borine serum, 1% Penicillin-Streptomycin solution, 50 ng/ml Granulocyte-macrophage colony-stimulating factor) (Stemcell technology, Canada). The medium was replaced every 2 days. On day 7, M0 Mφs were harvested for further experiments.

A total of 20 μl microparticles (Thermofisher, United States) diluted in 2000 μl 1% BSA were incubated at 37°C for 30 min and subsequent ultrasonic treatment for 5 min; then, 10^5^ Mφs were added to the microparticle solution and incubated for 1.5 h at 37°C. After centrifugation and washing, cells were diluted in 500 μl PBS and subjected to flow cytometry analysis at the fluoresceine isothiocyanate (FITC) wavelength (488 nm).

#### 2.7.6 Intestinal Organoids Culture

Small intestine tissue was washed with cold PBS15 times till it reached transparency and digested for 25 min (Stemcell technology, Canada). The supernatant was filtered and then centrifugated at 1,300 rpm for 5 min. The pellet was diluted in intestiCult organoid growth medium (Stemcell technology, Canada). The medium was exchanged every 2 days. On day 7, IOs were collected for further experiments.

#### 2.7.7 Immunofluorescence

For mitochondrial stress measurement, intestinal organoids were incubated with MitoSOX™ Red Mitochondrial Superoxide Indicator (ThermoFisher, United States) for 10 min at 37°C. After three times washing, cells were mounted on the fluorescent microscope at 590 nm.

To examine the protein abundance of BAX and BCL-2 in IOs, IOs were fixed by 4% PFA and treated with triton. After the blockage with 5% BSA, IOs were incubated with antibodies including BAX and BCL-2 (ThermoFisher, United States) overnight at 4°C. For CW-2 cells, cells were fixed and treated with blocking and permeabilization solution (10% FCS +0.1 Triton X100) and incubated with antibodies (vascular endothelial growth factor, VEGF; KI67; Vimentin) (ThermoFisher, United States) overnight at 4°C. After secondary antibody incubation, protein expression was visualized on the fluorescent microscope.

#### 2.7.8 Western Blotting

Sodium Dodecyl Sulphate-Polyacrylamide Gel Electrophoresis was performed utilizing 40 µg protein and transferred to a nitrocellulose membrane (VWR). After the blockage by 5% bovine serum albumin for 2 h, the membranes were then treated with antibodies for 24 h, including CD163, CD206, and ARG1 as well as GAPDH antibodies (1:1,000) (ThermoFisher). After secondary antibody incubation for 2 h, protein bands were visualized.

#### 2.7.9 Scratch Assay

Human colorectal cancer cells (CW-2) (American Type Culture Collection, United States) were seeded in DMEM complete medium (Biological industries, Israel) and the monolayer was scratched utilizing a pipette tip. The scratched area was photographically monitored at 0 and 24 h. The percentage of the coverage was measured.

#### 2.7.10 Transwell Assay and Co-culturing System

Cells were seeded in the upper chamber of transwell (Corning, 8 μm diameter) at a density of 10^5^ cells/per well, and a complete cell culture medium was put in the lower chamber. For co-culturing, M0 BMDMs were seeded in the upper chamber, and human colon epithelial cell cells (NCM460 cells) (Moyer et al., 1996) (American Type Culture Collection, United States) were put in the lower chamber of the insert with 1,640 complete medium (Biological industries, Israel).

After 24 h, the membrane of transwell insert was cut and fixed with 4% Paraformaldehyde Fix Solution (Beoytime, China) for 10 min. After washing with cold PBS, Dapi (Beoytime, China) was mounted on the membrane and visualized by microscope. The migrated cells were counted at five random fields and the average number was obtained.

#### 2.7.11 ELISA

Mouse IL-17, CXCL1, and Albumin Enzyme-Linked ImmunoSorbent Assay (ELISA) Kit were obtained from Abcam. Serum was incubated with antibody cocktail for 1 h. After three times washing, the supernatant was discarded and Streptacidin-horseradish peroxidase solution was added for 1 h. After incubating TMB (3,3′, 5,5; -tetramethylbenzidine) chromogen solution for 10 min, stop solution was utilized. OD was read at 450 nm.

## 3 Results

### 3.1 GCZX Capsule Suppresses Experimental Colitis Progression

**FIGURE 1:**
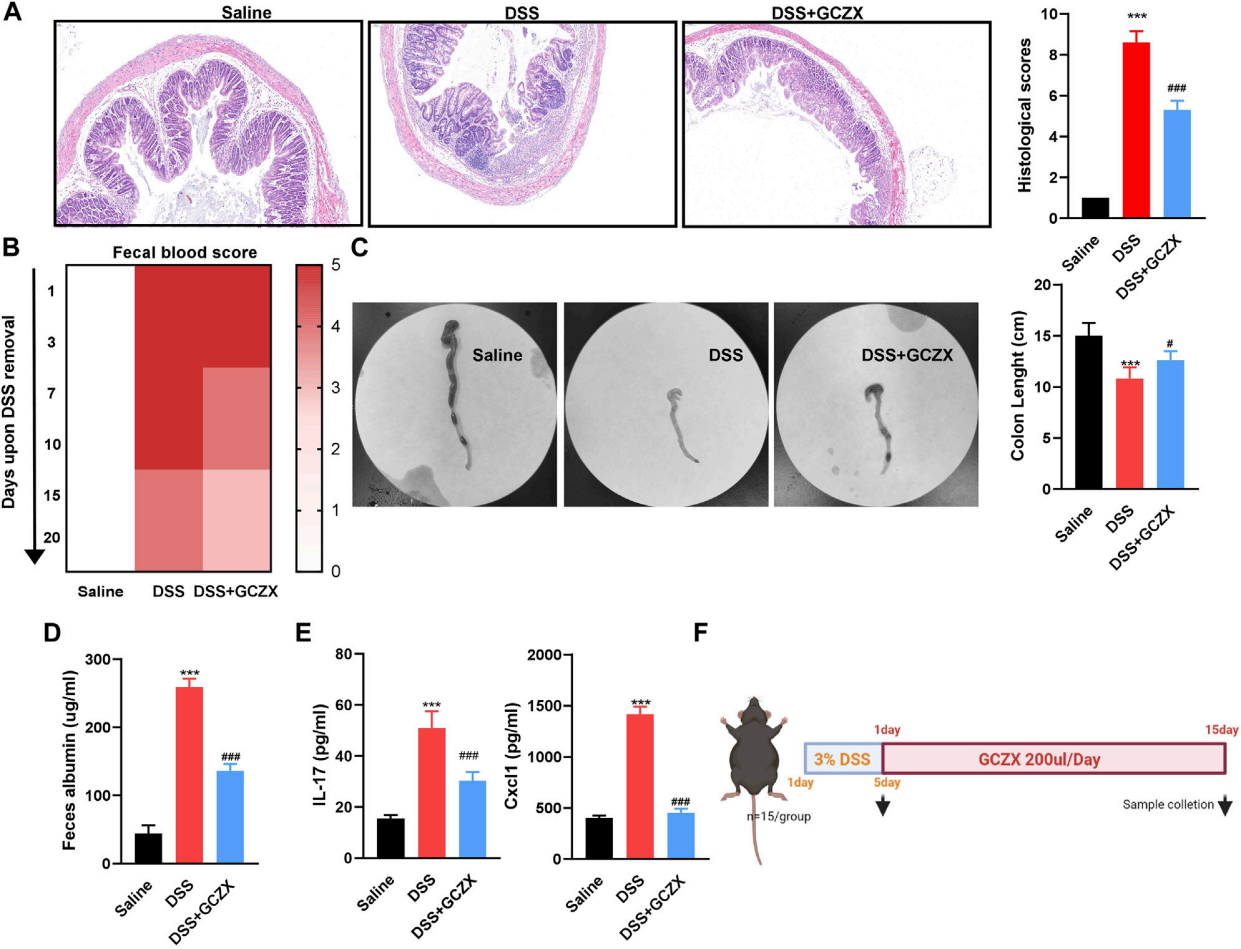
GCZX alleviates UC progression HE staining images of colon sections **(A)**, fecal occult blood test **(B)**, colon tissue **(C)**, albumin level **(D)**, IL-17 and CXCL1 levels **(E)** from C57 mice subject to 4% DSS administration with GCZX; Flow chart of experimental design **(F)**. *p < 0.05, **p < 0.01, and ***p < 0.001 indicates a statistically significant difference from saline group; # indicate statistically significant difference from UC group.

A murine model of experimental colitis was established utilizing C57 male mice that had free access to 4% DSS water for 5 days, and was gavaged twice a day with GCZX treatment (Figure 1F). HE staining showed that GCZX treatment dramatically relieved the epithelial structural collapse in UC mouse models (Figure 1A). Moreover, the severity of fecal blood and albumin, shortened colon length in UC mice were considerably alleviated after GCZX administration (Figures 1B–D); GCZX also reversed DSS-induced IL-17 and CXCL1 levels in mouse serum (Figure 1E), suggesting the intervention effect of GCZX capsule against experimental colitis.

### 3.2 HIT Network of GCZX

**FIGURE 2:**
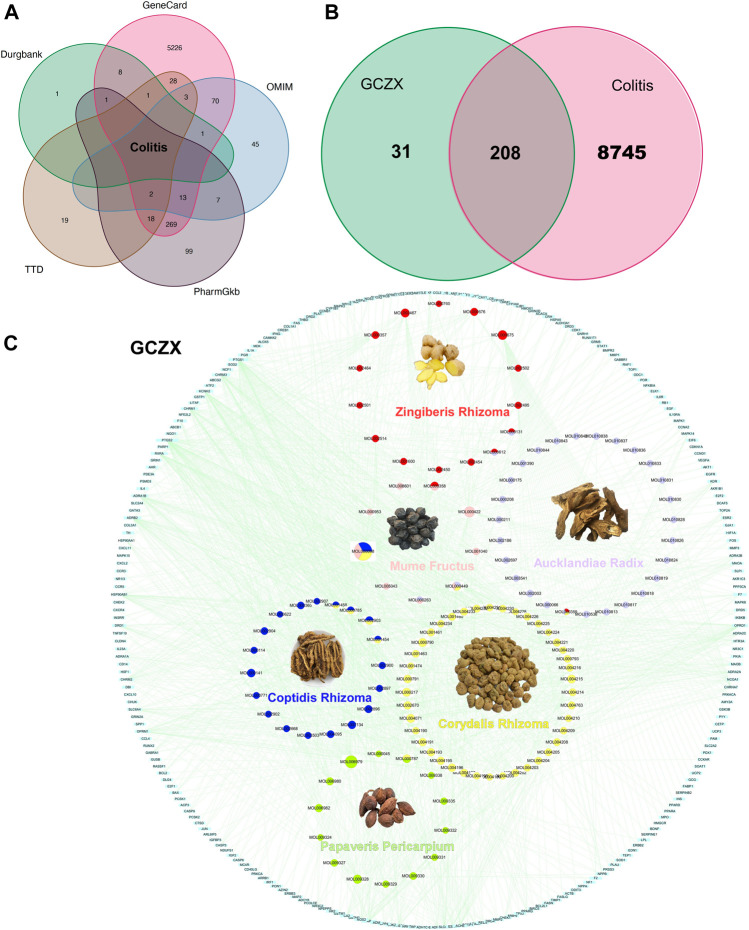
The Herb-Ingredient-Target network of GCZX. Venn analysis showing UC-related genes from five databases: Drugbank, GeneCard, OMIN, PharmGkb, TTD **(A)**, and the shared targets between GCZX and UC **(B)**; Network of herbs and compounds as well as all the potential targets **(C)**.

In virtue of the TCMSP database, we obtained 138 active compounds targeting 2,482 genes*.* ***Zr*** yields five components targeting 54 genes, ***CPr*** yields 26 components targeting 400 genes*,* Ar yields 35 components targeting 797 genes, ***Mf*** yields nine components targeting 308 genes, CRr yields 49 components targeting 164 genes, ***Pp*** yields 14 components targeting 374 genes (Supplementary Table S1). Based on the acquired 5811 UC-relevant genes (Figure 2A, Supplementary Table S2), Venn diagram demonstrated that GCZX formula shared 208 putative targets with UC (Figure 2B). With the aim of exploring the pharmacological mechanisms of GCZX, a HIT network was constructed (Figure 2C). The light blue rectangle nodes forming the outside circle represented UC-related genes and the circle nodes inside were GCZX active components; each color indicated one herb. As illustrated, all the herbs not only worked synergistically targeting some common genes but also shared some active components.

**FIGURE 3:**
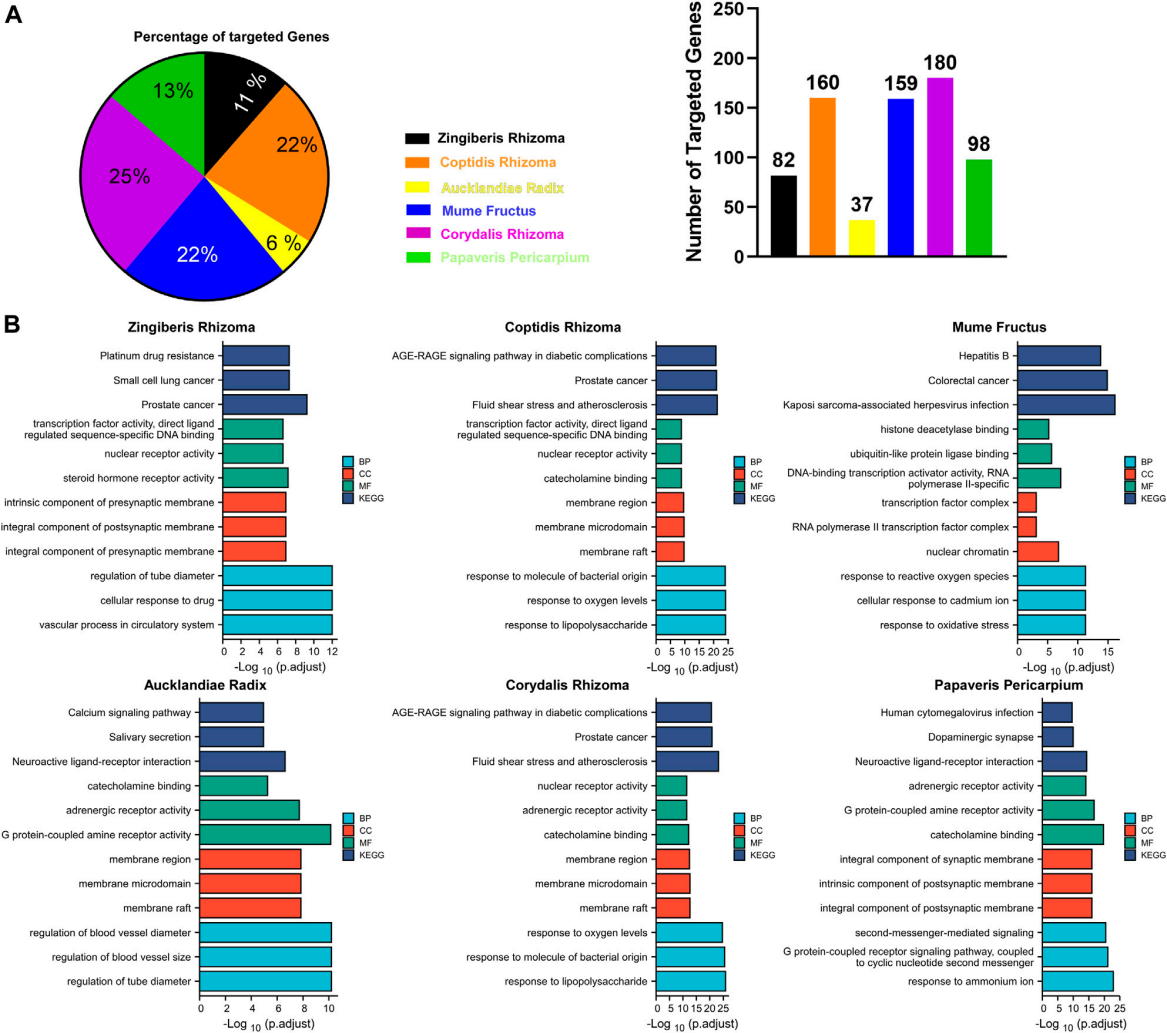
The function of each herb in the pathogenesis of UC. Pie chart illustrating the target genes of each herb in UC **(A)**; GO and KEGG analysis showing the enrichment of herb-target genes **(B)**.

As shown in Figure 3A, ***CRr***, ***CPr,*** and ***Mf*** were responsible for 69% of targeted genes and considered the major components of GCZX formula. GO and KEGG analysis was performed with these genes to predict the mechanism of each herb combating UC (Figure 3B). Mf, the core herb in the formula, correlated with the regulation of oxidative stress and response to reactive oxygen species that both play a pivotal role in the pathogenesis of IBD; and with the response to cadmium ion that causes microbiota dysbiosis hence increases intestinal permeability (Liu et al., 2020); and with histone deacetylase activity that is involved in tumorigenesis. Moreover, Mf influenced virus infection and the neoplastic progression of colorectal cancer. CRr and CPr shared many common components and their targeted genes were similarly enriched in immune response to lipopolysaccharide and bacterial as well as virus, all of which could induce inflammation and infection. Other seemingly minor but vital herbs were Ar and Zr as well as Pp. Pp and Zr as well as Ar worked with neurological processes, including neuroactive ligand-receptor interaction and neurotransmitter receptor activity as well as postsynaptic membrane, which pointed towards the modulation of intestinal innervation by these herbs. Additionally, *Ar*, *Pp*, *CRr,* and *CPr* were associated with catecholamine activity including dopamine and adrenaline, both of which innervate gastrointestinal muscles whereby regulating gastrointestinal motility. Specifically, Zr and Ar are also related to regulation of vascular processes such as blood vessel size and diameter, indicating a favorable role in hemostasis. Furthermore, GCZX represented a strong capacity to suppress neoplastic progression including prostate cancer, small cell lung cancer, and colorectal cancer. Altogether, these herbs function synergistically to alleviate rectal bleeding, orchestrate enteric nervous system, and control host immune response.

**FIGURE 4:**
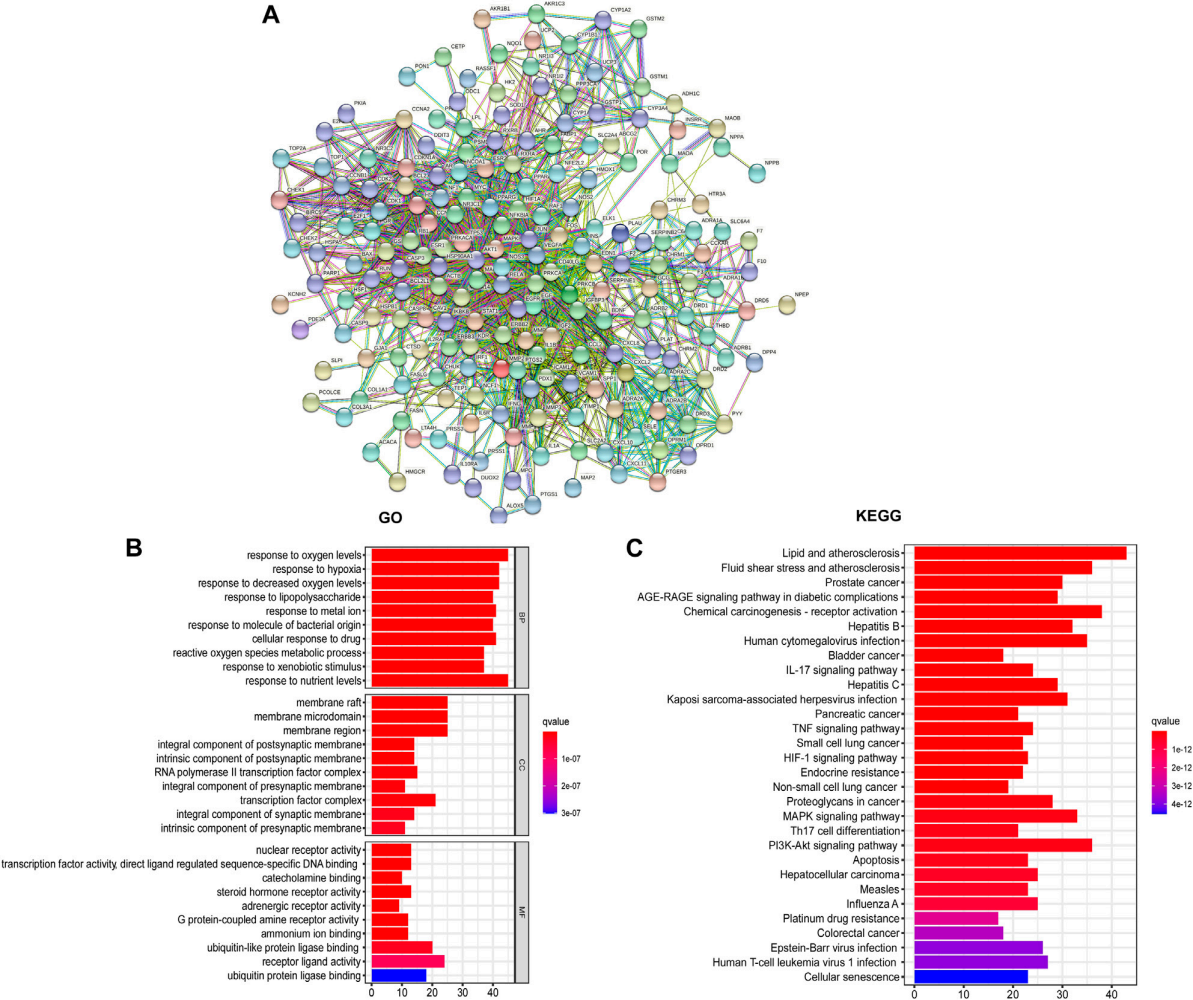
PPI network of GCZX targets. PPI interaction network established by the target genes of GCZX in UC (A); GO and KEGG analysis of GCZX targeted genes (B).

Utilizing the intersected genes between UC and GCZX, we construct a full PPI network with a PPI enrichment *p*-value (<1.0e-16) based on the STRING database (Figure 4A). In agreement with the individual function of each herb, GO analysis showed that these genes were enriched in responses to oxygen, hypoxia, lipopolysaccharide, bacterial, nutrient levels as well as cellular response to drug. KEGG further corroborated the role of GCZX in cancer, inflammation, and infection.

**FIGURE 5:**
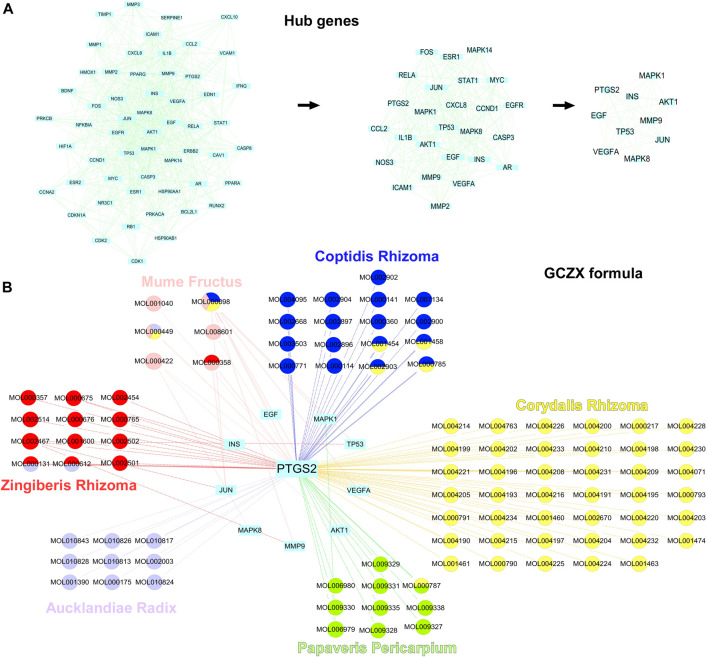
Hub-genes of PPI network. Hub genes of PPI interaction network **(A)**; Herbs and compounds as well as all hub-genes targets were linked **(B)**.

Sub-network was established with 10 hub-genes with higher than the median values of the indices (Figure 5A). The median values of betweenness centrality, closeness centrality, degree centrality, eigenvector centrality, local average connectivity, and network centrality in the PPI network were 291.9072, 0.408736, 15.35052, 0.049987, 6.280781, 8.980268, respectively. The median values in the final subnetwork (right) were 10.77105, 0.880509, 21.5, 0.223418, 14.83785, 18.95284, respectively. As shown in Figure 5B, we selected the common-shared components of the herbs for subsequent *in-vitro* experiments, composed of (R)-Canadine, palmatine, coptisine, palmatine, quercetin, beta-sitosterol, Stigmasterol, EIC (-)-alpha-cedrene, and fumarine. To validate the existence of these compounds and their metabolites, the serum isolated from DSS-GCZX mice was collected and subjected to LC-MS analysis (Supplementary Figure S1; Supplementary Table S3).

### 3.3 Fecal Metabonomic Combined With 16S rRNA Sequencing Validation

Network pharmacology analysis showed the antibacterial effect of GCZX capsule, so we examined changes in microbiota composition and its metabolites. With the aim of identifying whether the sequencing amount was sufficient to evaluate the diversity of the original microbiota, α-diversity was calculated by coverage meaning the true state of the microbes, Chao and Ace reflecting the bacterial richness and species abundance (Grice et al., 2009), and Shannon and Simpson indices reflecting the diversity of original microorganisms (White et al., 2009).

**FIGURE 6:**
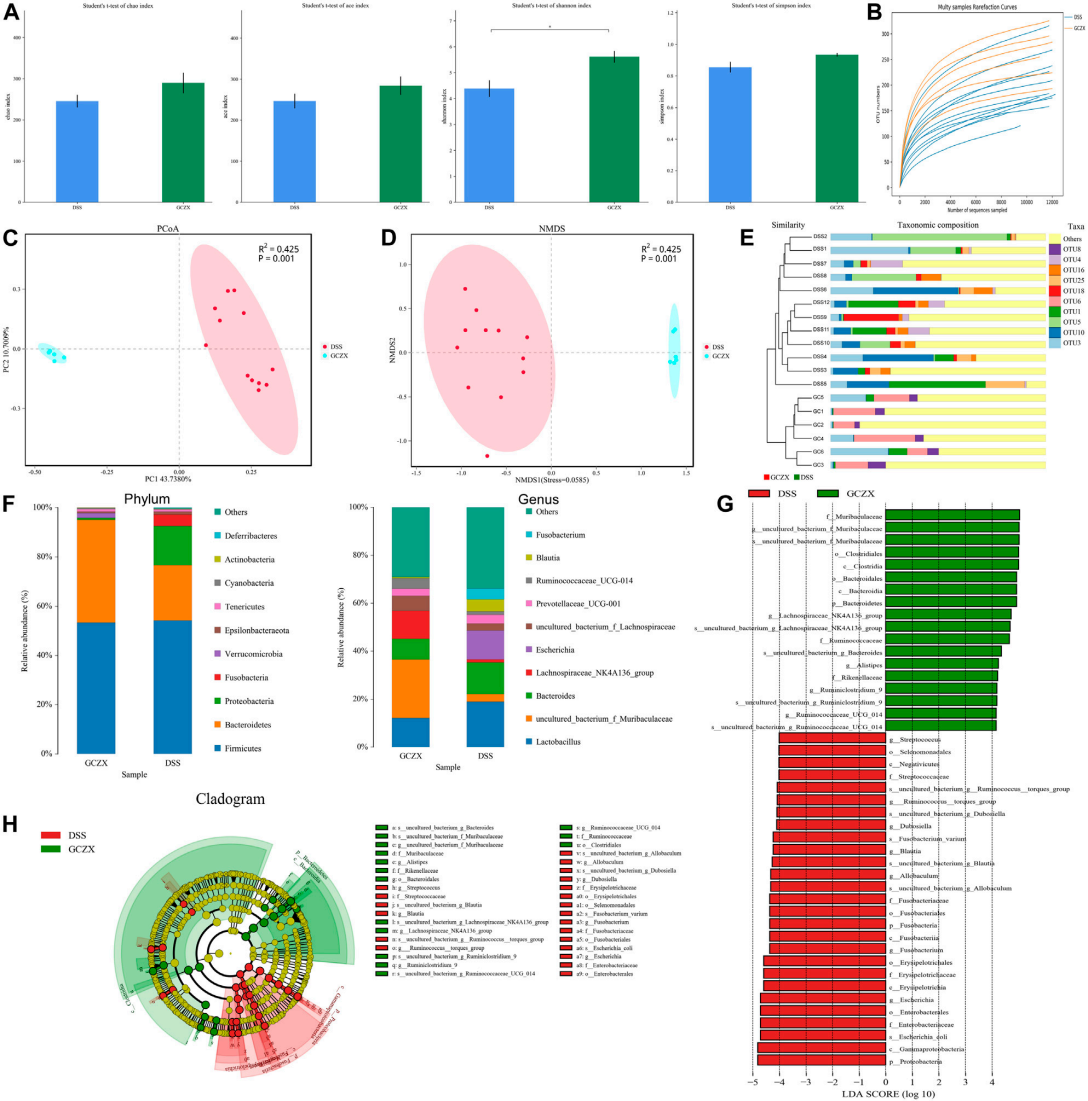
Profiles of microbial communities with GCZX treatment Altered Alpha-diversity of microbiota **(A)**, rarefaction analysis **(B)**, PoA **(C)**, and NMDS **(D)** as well as UPGMA **(E)** illustrating the clustering of gut flora between UC and GCZX-treated UC mice; Microbiota composition at the phylum and genus levels after GCZX therapy **(F)**; Cladogram **(H)** and LEFSe **(G)** illustrating the abundance of bacterial species between UC and GCZX-treated UC mice.

As illustrated in Table1, all sample libraries covered above 99%, which indicated the library size of this study sufficed to cover the majority of the microbes. Rarefaction analysis illustrated that the number of OTUs in both groups reached saturation (Figure 6B). Among the observed OTUs, GCZX treatment restored DSS-reduced bacterial diversity of UC mice without influencing the bacterial richness (Figure 6A). β-diversity reflecting between-habitat diversity was analyzed by binary Jaccard. Both PCoA and Nonmetric multidimensional scaling analysis (NMDS) indicated a clear distinction between DSS group and DSS-GCZX group (Figures 6C,D). Stress value <0.2 suggests the validity of NMDS. Furthermore, based on the distance matrix acquired from β-diversity, UPGMA was utilized to establish hierarchical clustering of these samples with the aim of evaluating the similarity of bacterial composition after GCZX therapy. As shown in Figure 6E, it manifested that DSS group and DSS-GCZX group clustered clearly in their own groups. Species annotation analysis showed only bacterial of the top 10 abundance at the genus and phylum levels with remaining species merged into “others.”

At the phylum level, the relative abundance of *Bacteroidetes* that belongs to normal gut microbiota (Le Chatelier et al., 2013) was higher in GCZX group, while *Fusobacteria* and *Proteobacteria*, reported as UC-related microorganisms (Petersen et al., 2020), were more abundant in the DSS group. At the genus level, the normal flora of the mouse gut such as Lachnospiraceae and Muribaculaceae (Chung et al., 2020) were most abundant in GCZX group (Figure 6F). Cladogram and Linear discriminal analysis coupled with effect size measures (LEFSe) revealed that *Ruminiclostrdium* and Lachnospiraceae, Muribaculaceae, and *Clostridia* were enriched in the GCZX group (Figures 6G,H).

**FIGURE 7:**
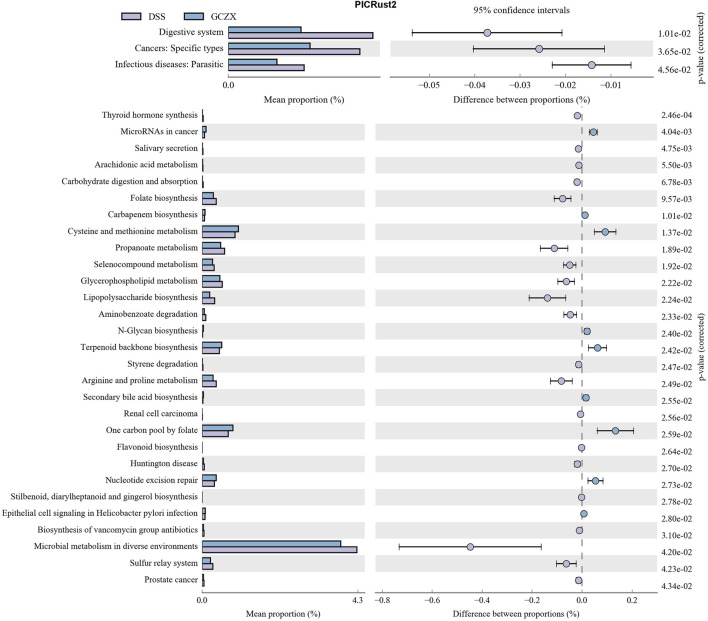
PICRust2 analysis of the therapeutic role of GCZX pellet.

Phylogenetic Investigation of Communities by Reconstruction of Unobserved States (PICRust2) (Langille et al., 2013) based on 16S rRNA sequencing data was utilized to predict functional profiles of microbial communities. GCZX treatment suppressed the incidence of cancer and infectious disease and digestive system (Figure 7). Specifically, GCZX inhibited microbial metabolism, suppressed UC-related metabolic pathways including arachidonic acid and glycerophospholipid metabolism (de Silva et al., 2010; Yuan et al., 2020) and abrogated pro-inflammatory responses such as lipopolysaccharide biosynthesis, restored colitis-reduced arginine (Coburn et al., 2016; Singh et al., 2019) and propanoate (Chambers et al., 2019) levels by impeding their metabolisms whereas contributed to methionine metabolism the derivatives of which favor reconstruction of destroyed epithelium during UC (Roediger et al., 1996; Stavsky and Maitra, 2019). Additionally, it facilitated antibiotics and secondary bile acid biosynthesis that both facilitate the remission of UC (Sinha et al., 2020). Tax4Fun (Aßhauer et al., 2015) also corroborated a strong inhibition of inflammatory pathways such as IL-17 and estrogen signaling pathways as well as antigen processing presentation, and the suppressed incidence of infectious diseases (Supplementary Figure S3).

### 3.4 GCZX Treatment Rescues TNFα-Induced ROS Production in Intestinal Stem Cells

As predicted in the network pharmacology analysis, the therapeutic mechanism of GCZX capsule against colitis involves oxidative stress that enables stem cells to take on an “over-active” state undergoing differentiation or apoptosis (Tothova et al., 2007; Zhang et al., 2018). The intestinal stem niche environment maintains homeostasis of epithelial renewal by orchestrating the balance between stemness and differentiation. IO culture directly resembles intestinal epithelial organization and thereby recapitulates the dynamic cellular processes of re-epithelization under pathological settings, such as the stimulation of pro-inflammatory cytokines (TNFα).

**FIGURE 8:**
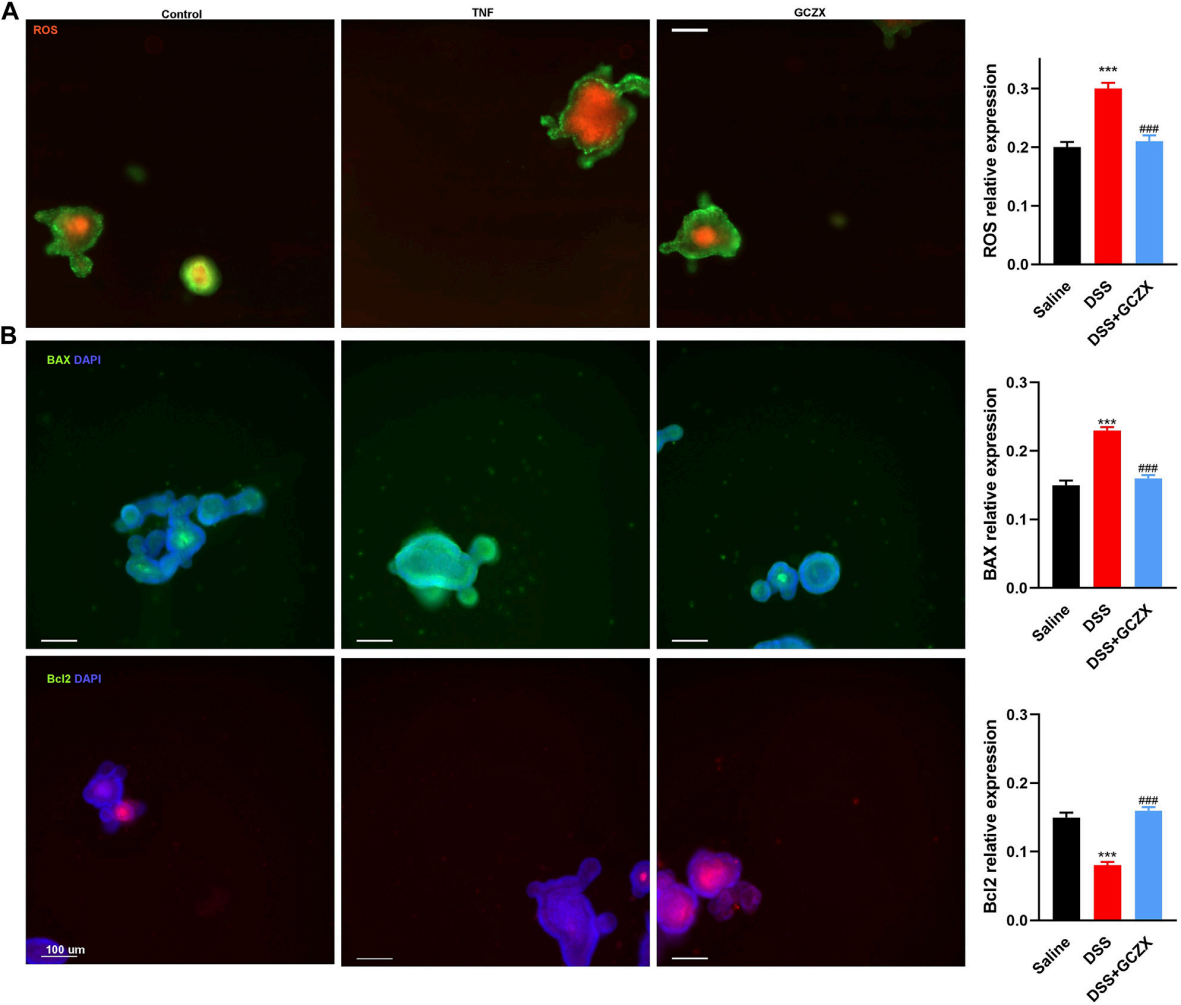
GCZX treatment alleviates TNFα-induced apoptosis of intestinal organoids. Immunofluorescent staining illustrating the mitochondrial stress **(A)** and BAX as well as BCL-2 expression **(B)** of TNFα-treated (20 ng/ml, 24 h) intestinal organoids in the presence of GCZX (30 ng/ml, 24 h). *p < 0.05, **p < 0.01, and ***p < 0.001 indicates a statistically significant difference from saline group; # indicate statistically significant difference from UC group.

An *ex-vivo* UC cellular model was constructed utilizing IOs in the presence of TNFα (20 ng/ml, 24 h), and GCZX solution was administered for another 24 h. Mitochondria consume oxygen and produce reactive oxygen species (ROS), which can be detrimental resulting in intrinsic apoptosis or initiate acute responses to external stimuli such as pro-inflammatory interleukins and pathogens (El-Osta and Circu, 2016). As illustrated in Figure 8A, TNFα treatment enhanced IO ROS production and inflated its size, an effect that was restored by GCZX administration. In line with the findings above, immunofluorescence showed TNFα-induced apoptosis was suppressed by GCZX therapy (Figure 8B). Therefore, GCZX treatment could suppress apoptosis of intestinal stem cells, thereby favoring mucosal healing and combating UC progression.

### 3.5 GCZX Therapy Inhibits Inflammation

**FIGURE 9:**
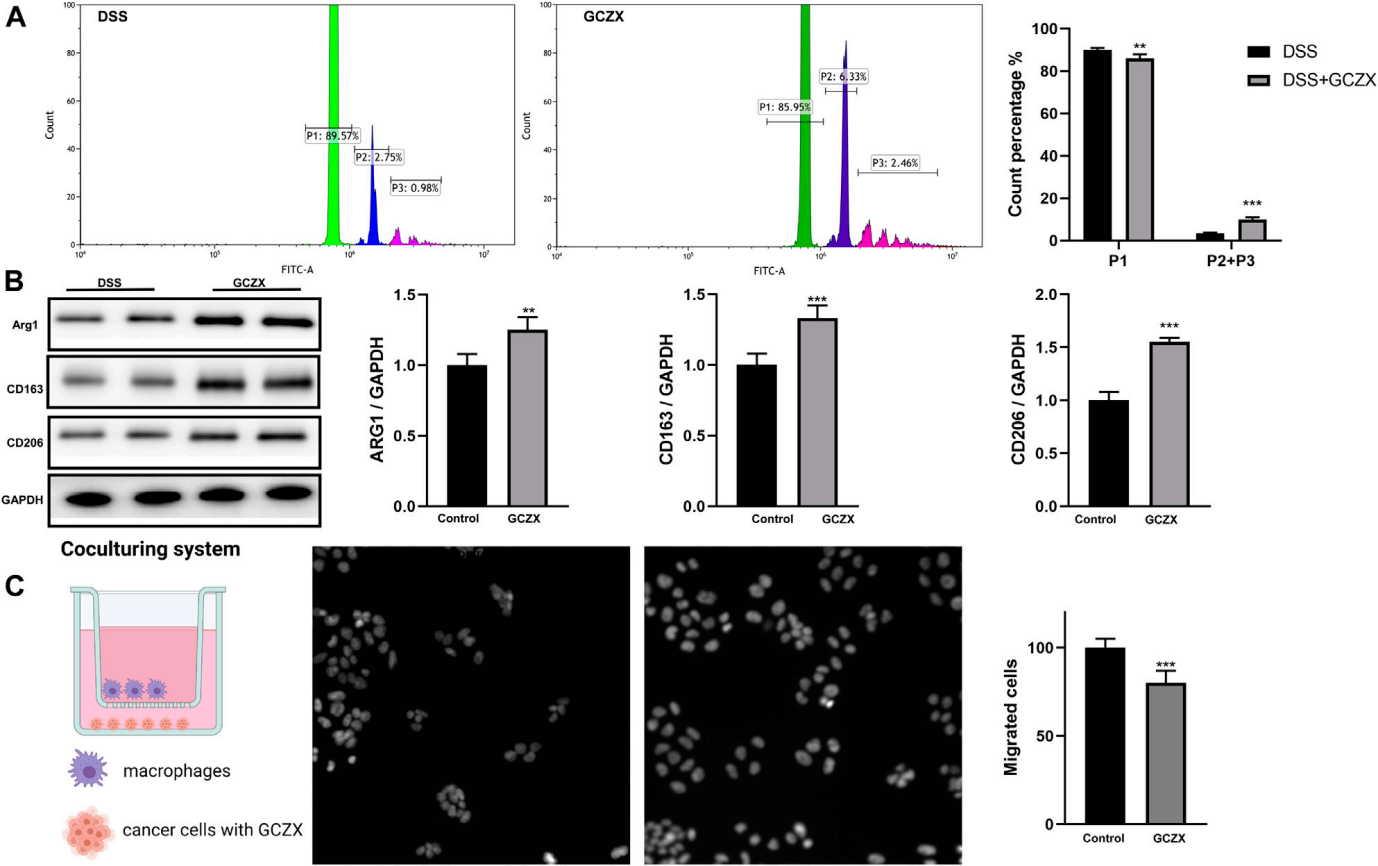
The reciprocal interaction between epithelial cells and immune cells Bar charts (n = 5) illustrating the phagocytic capacity of macrophages, green represents one microparticle engulfment (P1), blue represents two microparticles (P2), red represents more than two microparticles (P3) **(A)**; Western blotting showing the protein abundance of M2 macrophage markers **(B)**; Transwell assay showing the migration of macrophages incubated with GCZX-pretreated NCM460 cells (C). *p < 0.05, **p < 0.01, and ***p < 0.001 indicates a statistically significant difference from saline group; # indicate statistically significant difference from UC group.

Peritoneal Mφs isolated from GCZX mice showed stronger phagocytic capacity and higher expression levels of M2 markers compared with DSS group, as evidenced by flow cytometry and western blot (Figures 9A,B), corroborating the anti-inflammatory effect of GCZX capsule.

To mimic the microenvironment in proximity to epithelial cells, we established a co-culture system utilizing BMDMs and NCM460 cells. Interestingly, the migration of BMDMs was subdued with GCZX-pretreated NCM460 cells, indicating that GCZX treatment weakens chemokine secretion from NCM460 cells and hence inhibits the recruitment of BMDMs (Figure 9C).

Given the anti-tumor role of GCZX capsule predicted by network analysis, we examined the metastatic expansion of disseminated cancer cells in the presence of GCZX solution. KI67 represents proliferation, and VEGF and Vimentin refer to the invasion of cancer cells. Neither migration nor invasion as well as proliferation of CW-2 cells was affected by GCZX treatment, as shown by scratch assay and ICC experiments (Supplementary Figure S4), which suggested that GCZX capsule might influence tumor microenvironment, but not directly suppress tumor invasion.

## Discussion

IBD is a chronic remitting gastrointestinal disease characterized by impaired intestinal homeostasis as well as abnormal stress response to stimuli. Despite large-scale functional genomics findings regarding its pathogenesis, it is still relapsing in nature with uncertain etiology. The uncertainty relates to a broad array of pathological factors such as genetic susceptibility, environment, microbiota and food intake, etc. In this context, single-gene manipulation against IBD does not achieve the desired outcome nor does it without side effects (Hopkins, 2008). Multi-target drug strategy, comparatively, appears a promising direction to combat IBD by deploying multiple mechanisms. Moreover, emerging network pharmacology that integrates systems biology and network analysis and connectivity provide a practical approach to investigate the pharmacological mechanisms of traditional Chinese formulas (TCM) that encompass multiple compounds with proven efficacy for hundred decades in China.

GCZX capsule is derived from Wumei (Mume Fructus) pellet and has been included in the Chinese Pharmacopoeia 2020 to treat chronic gastrointestinal disorders. In the present study, by using a murine DSS-induced colitis model, we confirmed that GCZX formula dramatically hampered the progression of experimental colitis. Moreover, network pharmacology analysis showed the enrichment of GCZX-targeted genes in infection, inflammation, and cancer pathways by regulating the responses to oxygen, hypoxia, and pro-inflammatory molecules, and in hormone activities, which logically indicates the therapeutic effect of GCZX formula on UC. An effective UC intervention should take the key symptoms into consideration, namely, inflammation, rectal bleeding, mucosal healing, electrolyte turbulence, as well as mental suffering.

In this formula, ***Mf***, ***CPr***, ***CRr,*** and ***Pp*** synergistically exerted an anti-inflammatory function and rendered tissue protection from microbe-induced infection, by modulating responses to external stimuli including bacterial, lipopolysaccharide, etc. Concurrently, the combination of ***Ar*** and ***Zr*** functioned as hemostasis components by regulating blood vessel diameter and vascular processes. In terms of humoral regulation, ***Pp***, ***CPr***, ***CRr***, ***Ar,*** and ***Zr*** were associated with catecholamine, dopamine, as well as steroid hormone activities, which play a role in orchestrating the homeostasis of gastrointestinal tract and in relieving mental stress (Nezi et al., 2000; Furlan et al., 2006; Mogilevski et al., 2019). Moreover, GCZX formula took the brain-gut axis into consideration and selected compounds able to influence neurotransmitter activity. GO and KEGG analysis showed that ***Zr***, ***Ar,*** and ***Pp*** were involved in neurological processes, including neuroactive ligand-receptor interaction and synaptic membrane structure. Additionally, GCZX capsule showed an anti-cancer effect against colorectal cancer, prostate cancer, and small cell lung cancer, and also solved the resistance problem of anti-tumor drugs, such as platinum drugs. Collectively, network pharmacological analysis predicted that GCZX formula provides an effective therapeutic strategy against UC by abrogating infection and inflammation, restoring homeostasis of hormones and innervation, timely stopping bleeding, and relieving mental suffering. In a bid to validate the prediction, we assessed the molecular mechanisms via which GCZX capsule hinders UC progression and abrogates subsequent neoplasias. Herewith, its therapeutic effects on bacterial flora, host immunity, and mucosal healing were examined, respectively.

Human gut microbiota, comprised of over 35,000 bacterial species, is largely responsible for normal individual and human health as a whole by providing resistance against colonization of exogenous pathogens. Disruption of colonization resistance due to microbiota dysbiosis, and potential subsequent bacterial enteric infection is an important cause of gastrointestinal diseases. The decreased bacterial diversity in IBD was reversed after GCZX administration. Specifically, GCZX therapy restored the relative abundance of *Bacteroidetes*, Lachnospiraceae, and Muribaculaceae (Chung et al., 2020) that predominantly reside in healthy human gut and contribute to colonization resistance. Concomitantly, among the most prevalent bacterial enteropathogens (Ducarmon et al., 2019; Petersen et al., 2020), UC-associated *Fusobacteria* and *Proteobacteria* abundance was suppressed by GCZX treatment. In the formula, the active components of ***CRr*** and ***CPr*** show a strong anti-microbe activity (Wang and Ng, 2001; Li et al., 2005; Kim et al., 2014; Tian et al., 2020) and suppress subsequent inflammatory responses (Kubo et al., 1994; Wang and Ng, 2001) and neoplasias (Peng et al., 2006; Chen et al., 2016; Wan et al., 2019). Moreover, a healthy gut flora maintains the integrity of gut mucosal barrier and secrets antibacterial components, and provides nutrients and energy by metabolizing dietary components, and these beneficial effects have been disturbed during IBD.

As predicted by Tax4Fun and PICRust2 analysis based on profiles of microbiota composition, GCZX treatment suppresses UC-related metabolic pathways and inhibits pro-inflammatory responses, enhanced the production of antibiotics and secondary bile acid that is in favor of colonization resistance during UC remission (Ducarmon et al., 2019; Sinha et al., 2020). Collectively, GCZX capsule reduced the incidence of pathogen-induced infection by restoring a healthy gut flora that co-evolves to live in a collaborative relationship with host immunity.

We isolated peritoneal Mφs from the experimental colitis mouse model and examined the polarization of these cells. Upon stimuli, naïve macrophages (M0 Mφs) transit to pro-inflammatory phenotype (M1 Mφs) or anti-inflammatory status (M2 Mφs) depending on the types of external signals. GCZX capsule increased the phagocytic capacity of peritoneal Mφs and the corresponding protein expression of M2 Mφ makers, suggesting an alleviated inflammatory together with an improved would healing circumstance due to M2 Mφ transition. Moreover, within the inflammatory milieu, gut mucosal epithelial cells secret chemokines and subsequently attract a wide array of immune cell types. The secreted molecules could shape the plasticity of these recruited immune cells, such as Mφ polarization, while activated immune cells in turn influence differentiation and proliferation of mucosal cells, forming a reciprocal interaction. We found that GCZX-treated human colon epithelial cells showed a weaker capacity to attract M0 Mφ in comparison to non-treated cells, indicating that GCZX might reduce chemokine secretion of colon cells. Altogether, in the context of immunomodulation, GCZX alleviates colitis progression by hampering Mφ infiltration and suppressing its transition to a pro-inflammatory phenotype.

Given the suppressed inflammatory responses and restored gut microbiota after GCZX treatment as well as its anti-oxidative function demonstrated in network analysis, we ascertained whether it contributes to mucosal healing, a process that would be destroyed by any disruption in intestinal stem cell (ISC) niche and subsequently causes gastrointestinal diseases (Merlos-Suárez et al., 2011; Suzuki et al., 2018; Yu et al., 2020). Proper functions of ISCs require fine-tuning of cellular pathways involved in differentiation, proliferation, and underlie mitochondrial homeostasis. Mitochondrial are key organelles in that they provide energy and regulate cellular processes ranging from signaling to apoptosis, which is coupled with the production of ROS (Zorov et al., 2014). High levels of mitochondrial ROS can be detrimental resulting in intrinsic apoptosis (El-Osta and Circu, 2016) and hence impair ISC functions, and consequently hinder mucosal healing. As a primary culture of intestinal stem cells, IOs directly recapitulate the dynamic progression of mucosal destruction during IBD. In a bid to mimic the inflammatory setting in proximity to stem cell niche, mitochondrial stress in IOs was examined in the presence of TNFα. Expectedly, TNFα instigated ROS production of IOs and subsequently induced apoptosis, which was markedly alleviated by GCZX treatment, suggesting its beneficial role in mucosal renewal.

## Conclusion

**FIGURE 10:**
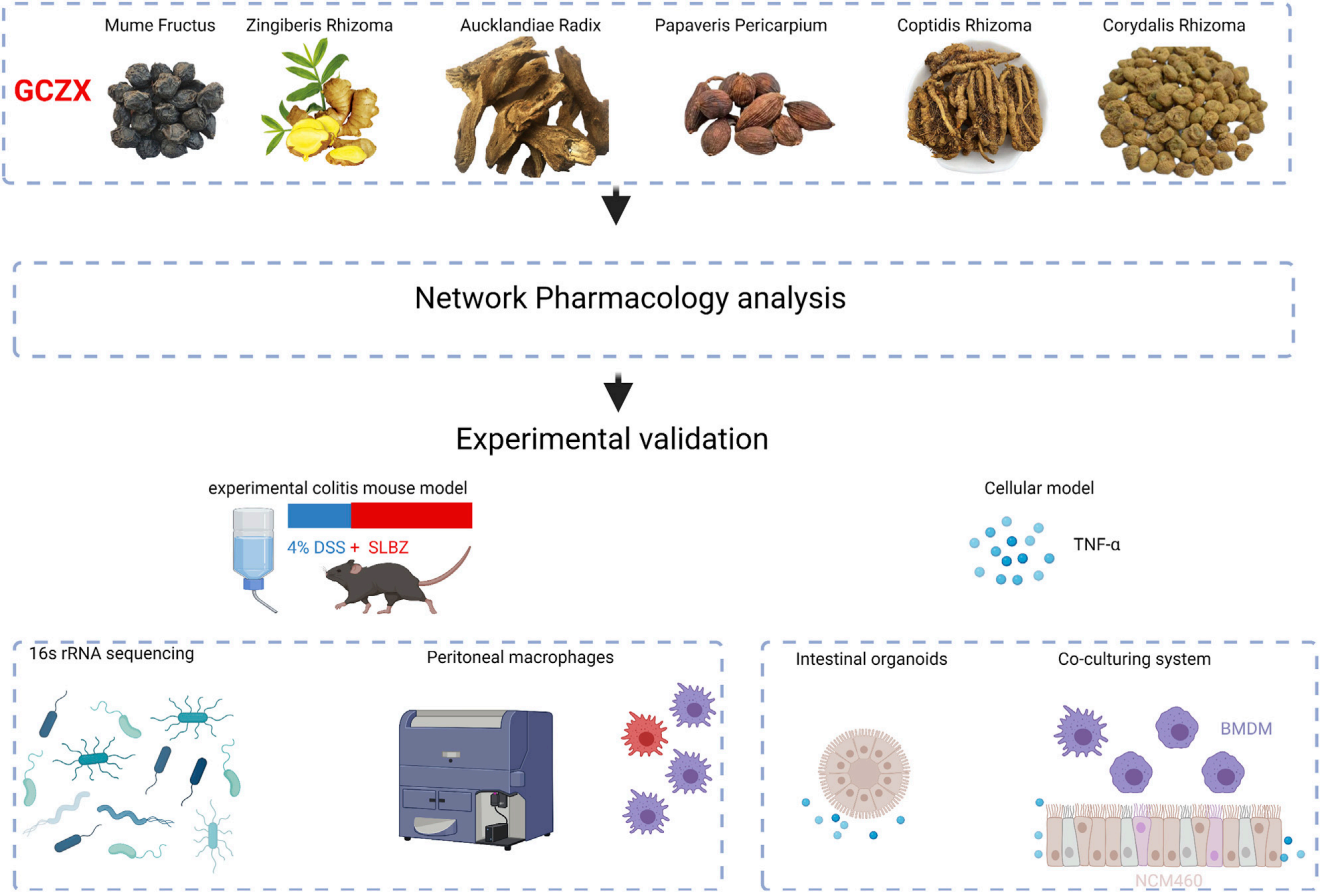
Shematic flow of the present study.

Based on the network pharmacology approach integrated with 16S rRNA sequencing and cellular evidence (Figure 10), we proposed that GCZX capsule inhibits the progression of experimental colitis by restoring healthy microbiota composition, suppressing inflammation and oxidative stress, and improving mucosal healing, which is an effective drug for colitis with proven safety.

## Data Availability

The datasets presented in this study can be found in online repositories. The names of the repository/repositories and accession number(s) can be found in the article/Supplementary Material.

## Abbreviations

CD, Crohn’s disease; GCZX, *Guchangzhixie*; IBD, Inflammatory bowel disease; IOs, intestinal organoids; Mφs, Macrophages; ISCs, intestinal stem cells; TCM, Traditional Chinese Medicine; PPIs, Protein-protein interactions; UC, Ulcerative colitis.
