# Supplementary figures and images for: The Pharmacological Mechanism of Guchangzhixie Capsule Against Experimental Colitis
Source: Front Pharmacol. 2021 Nov 18;12:762603. doi: 10.3389/fphar.2021.762603 (PMC8637769; doi:10.3389/fphar.2021.762603)

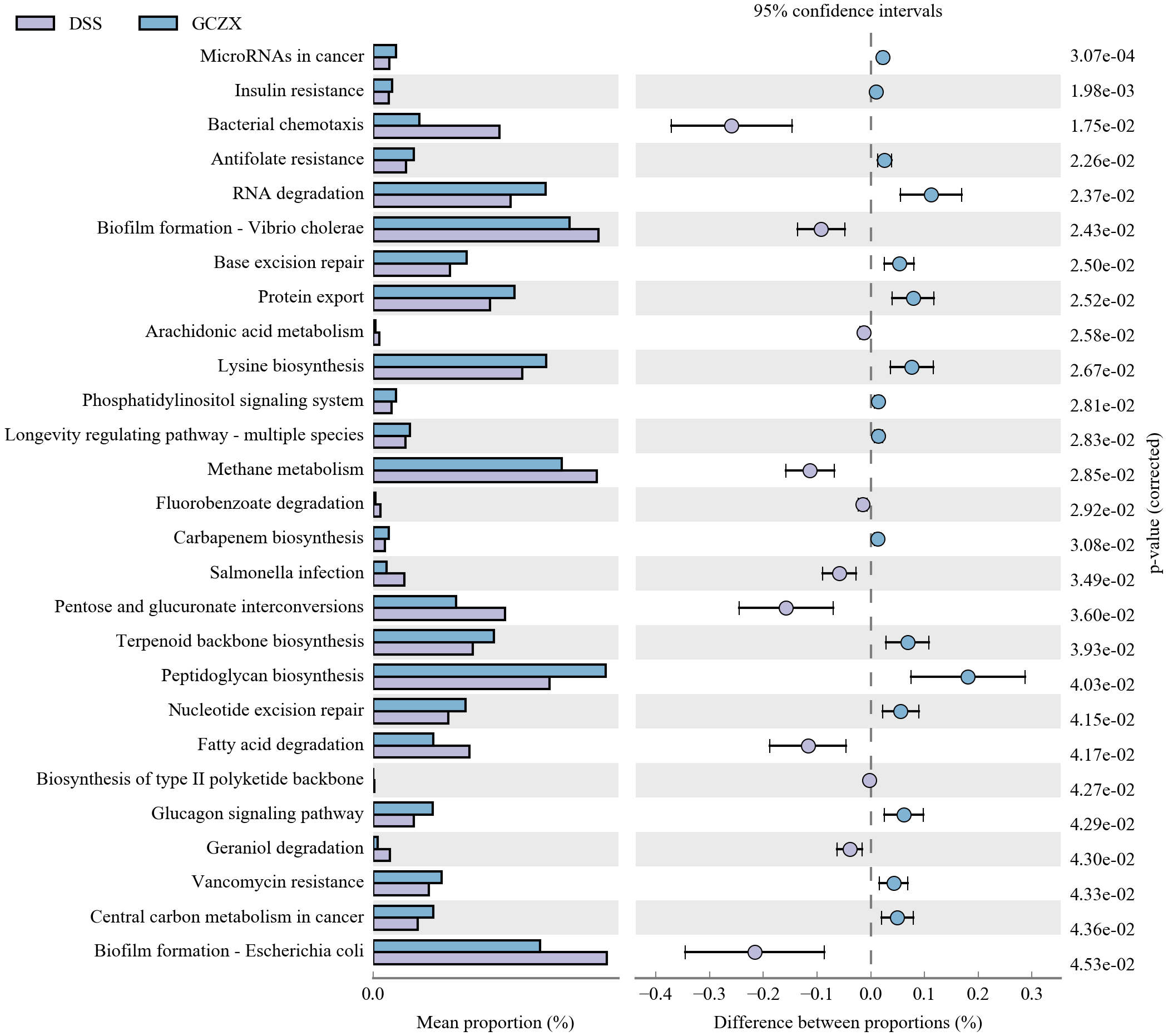

Supplement: Supplementary file 2 [file Image3.JPEG]

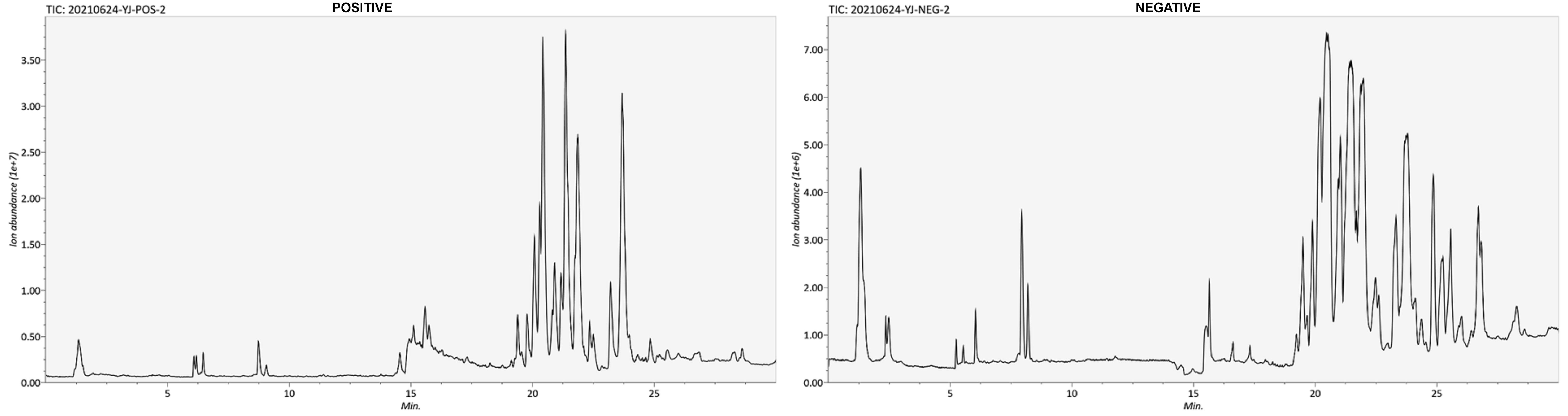

Supplement: Supplementary file 5 [file Image1.JPEG]

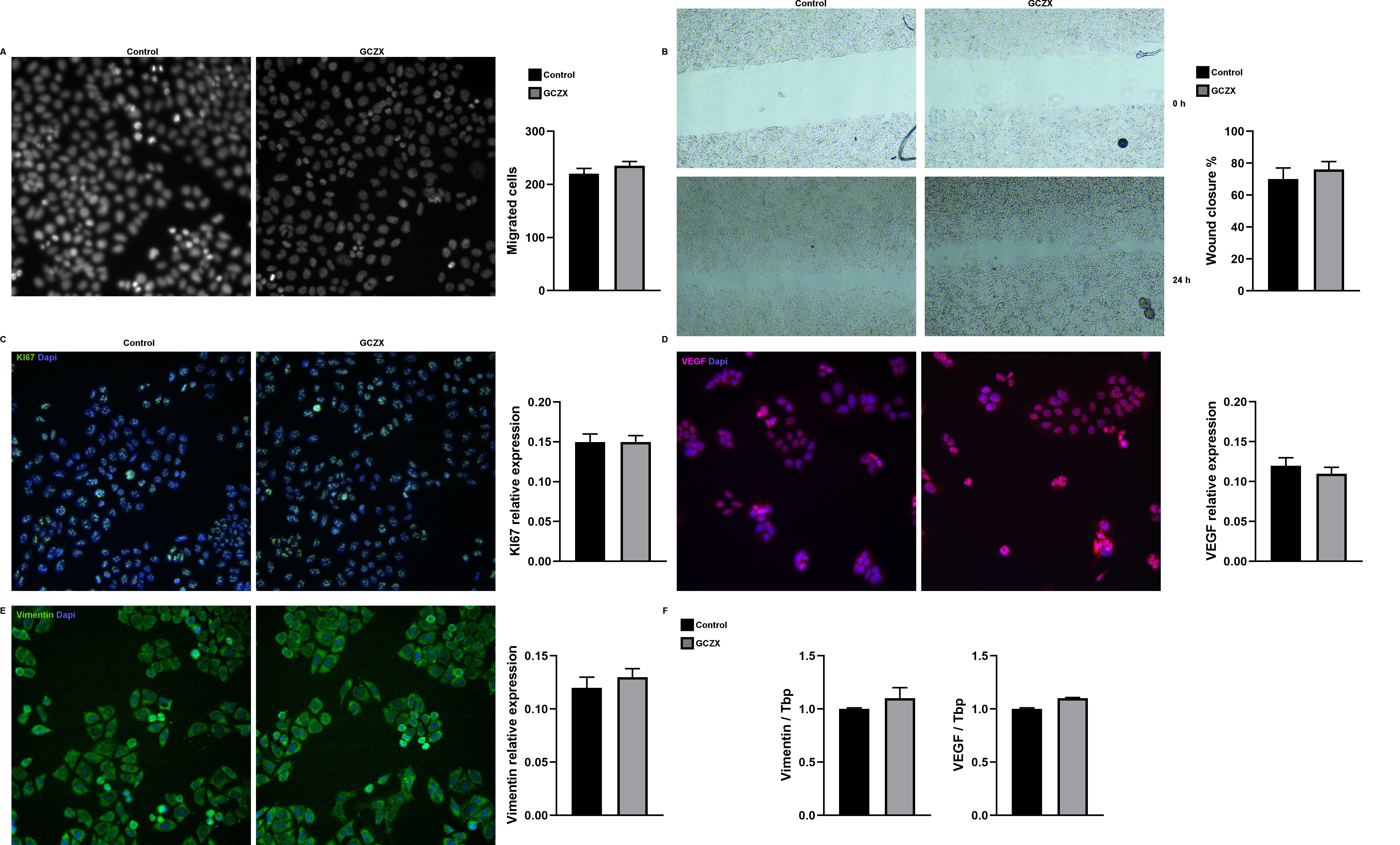

Supplement: Supplementary file 6 [file Image4.JPEG]

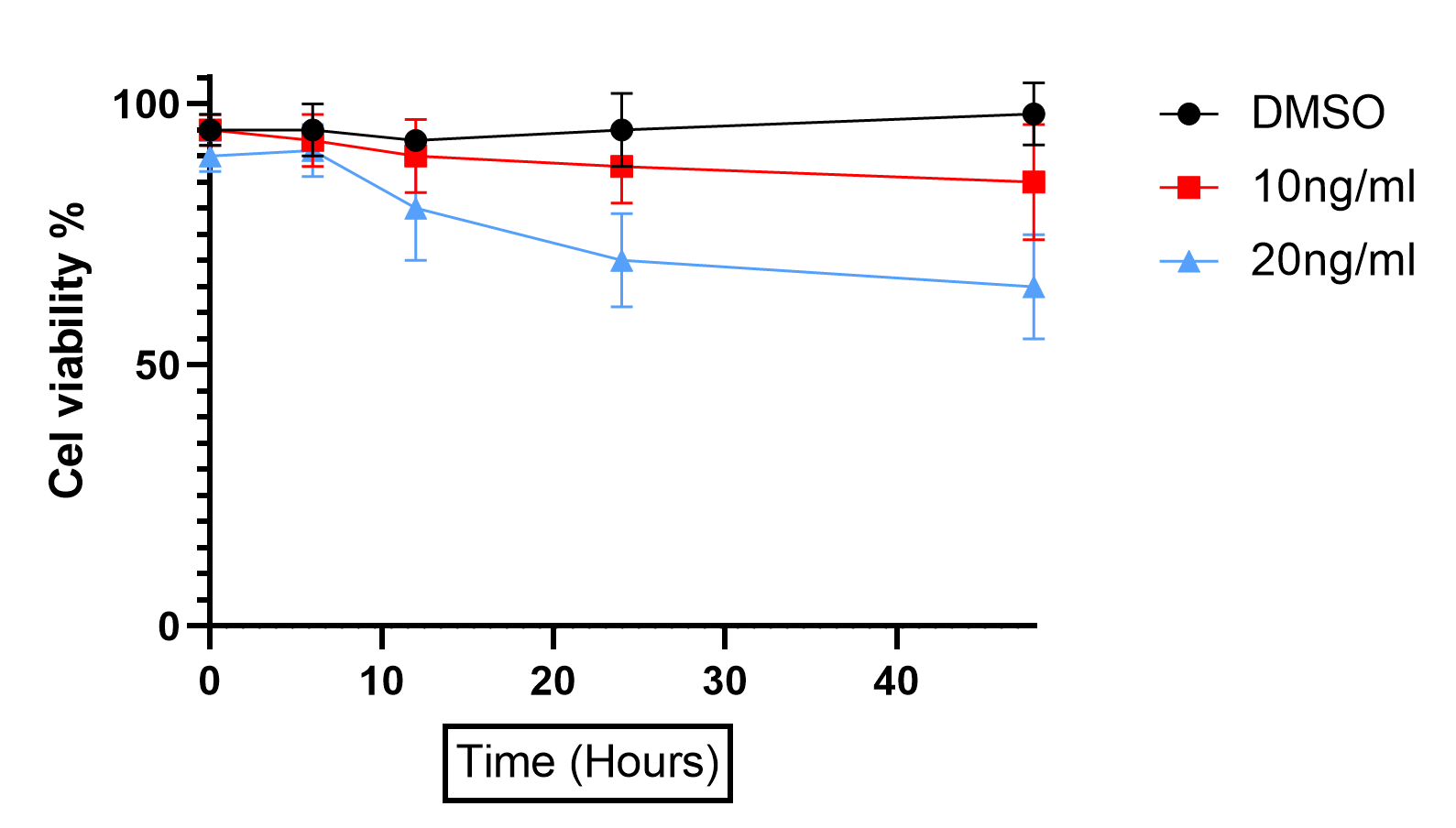

Supplement: Supplementary file 9 [file Image2.PNG]

## Slide 1
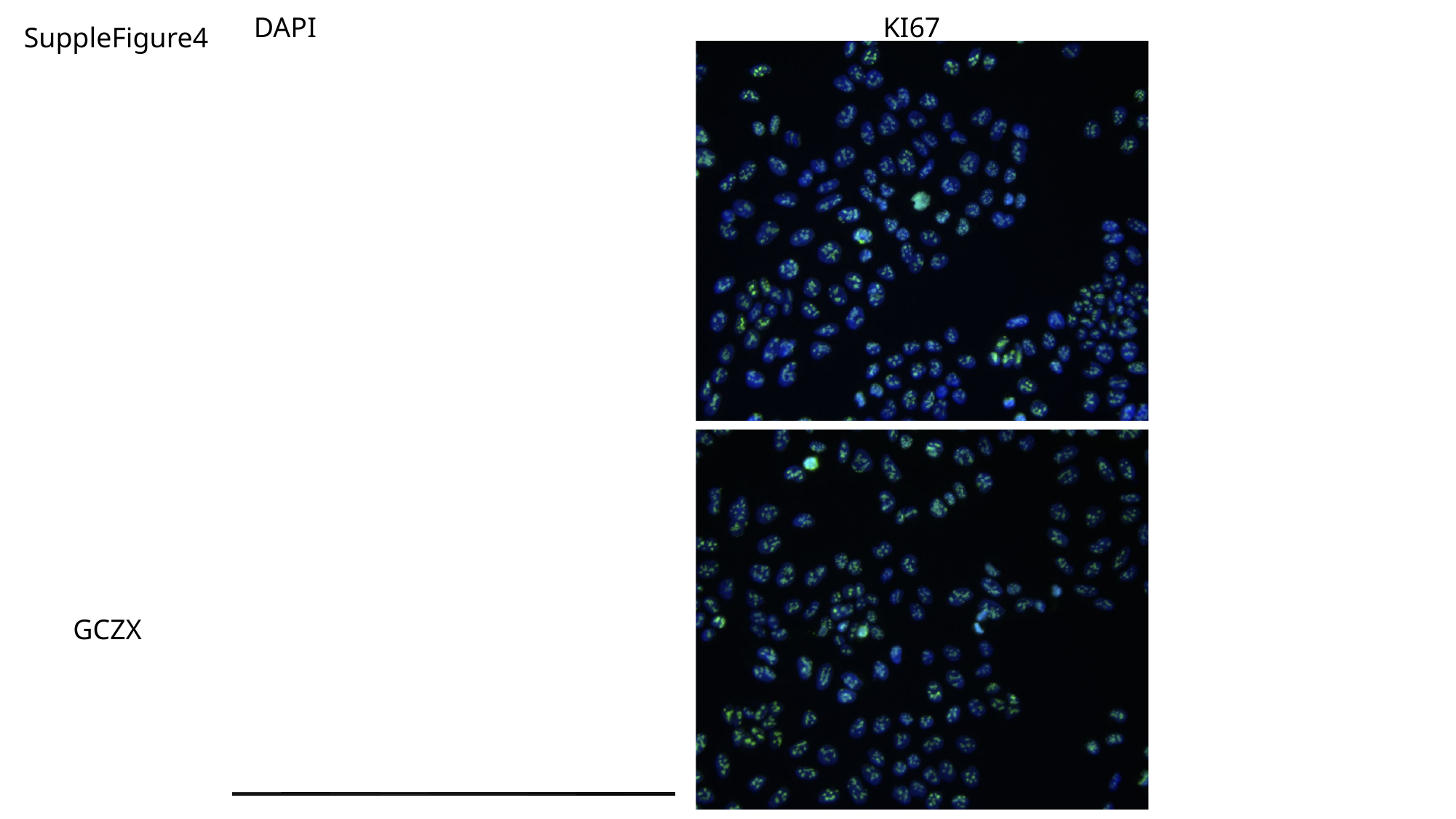

DAPI
KI67
SuppleFigure4
GCZX

## Slide 2
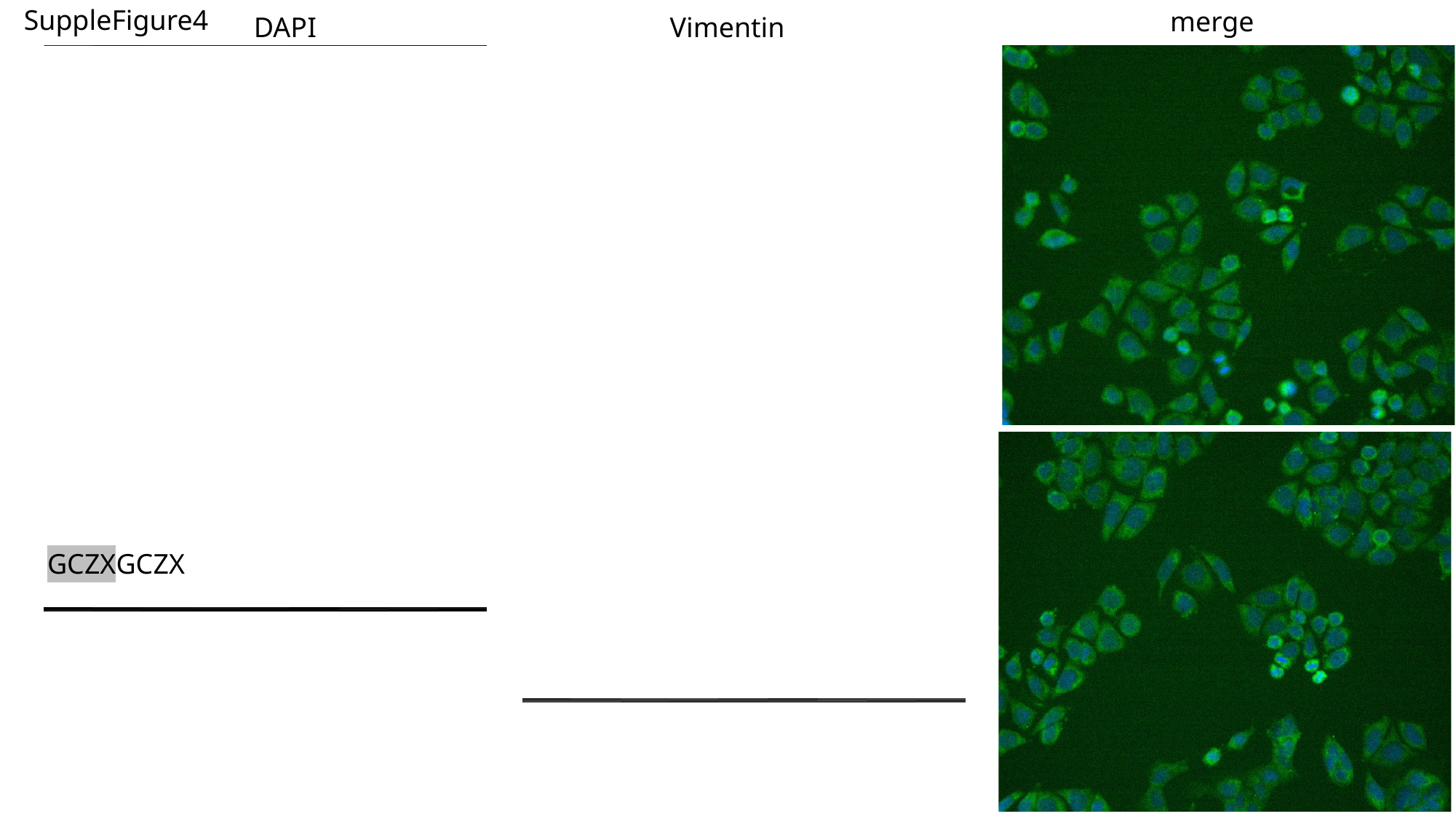

SuppleFigure4
merge
DAPI
Vimentin
GCZXGCZX

Supplement: Supplementary file 11 [file Presentation2.PPTX]
