# Supplementary material for: The Pharmacological Mechanism of Guchangzhixie Capsule Against Experimental Colitis
Source: Front Pharmacol. 2021 Nov 18;12:762603. doi: 10.3389/fphar.2021.762603 (PMC8637769; doi:10.3389/fphar.2021.762603)
Supplement: Supplementary file 4 [file Presentation1.PPTX]

## Slide 1
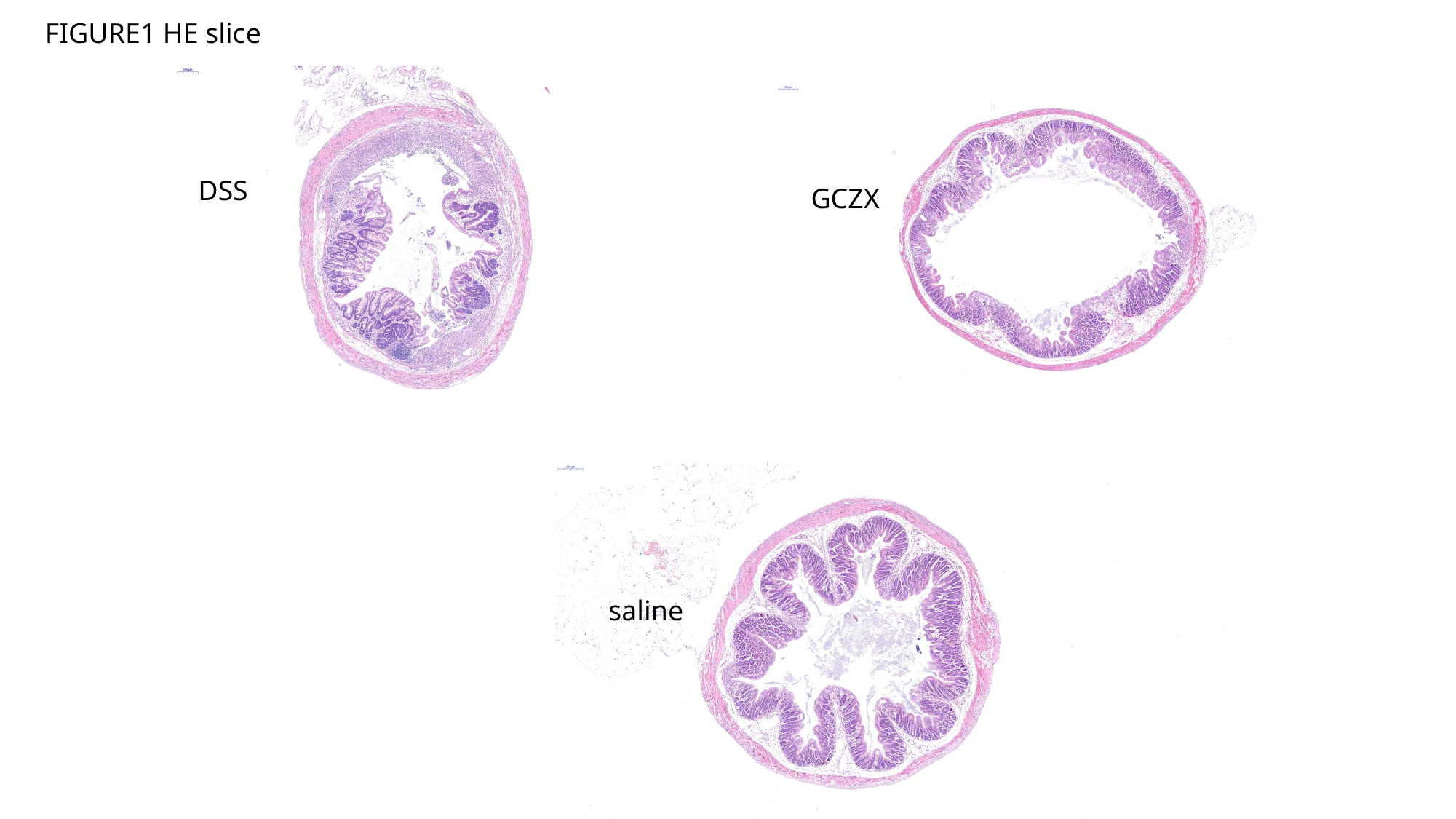

FIGURE1 HE slice
DSS
GCZX
saline

## Slide 2
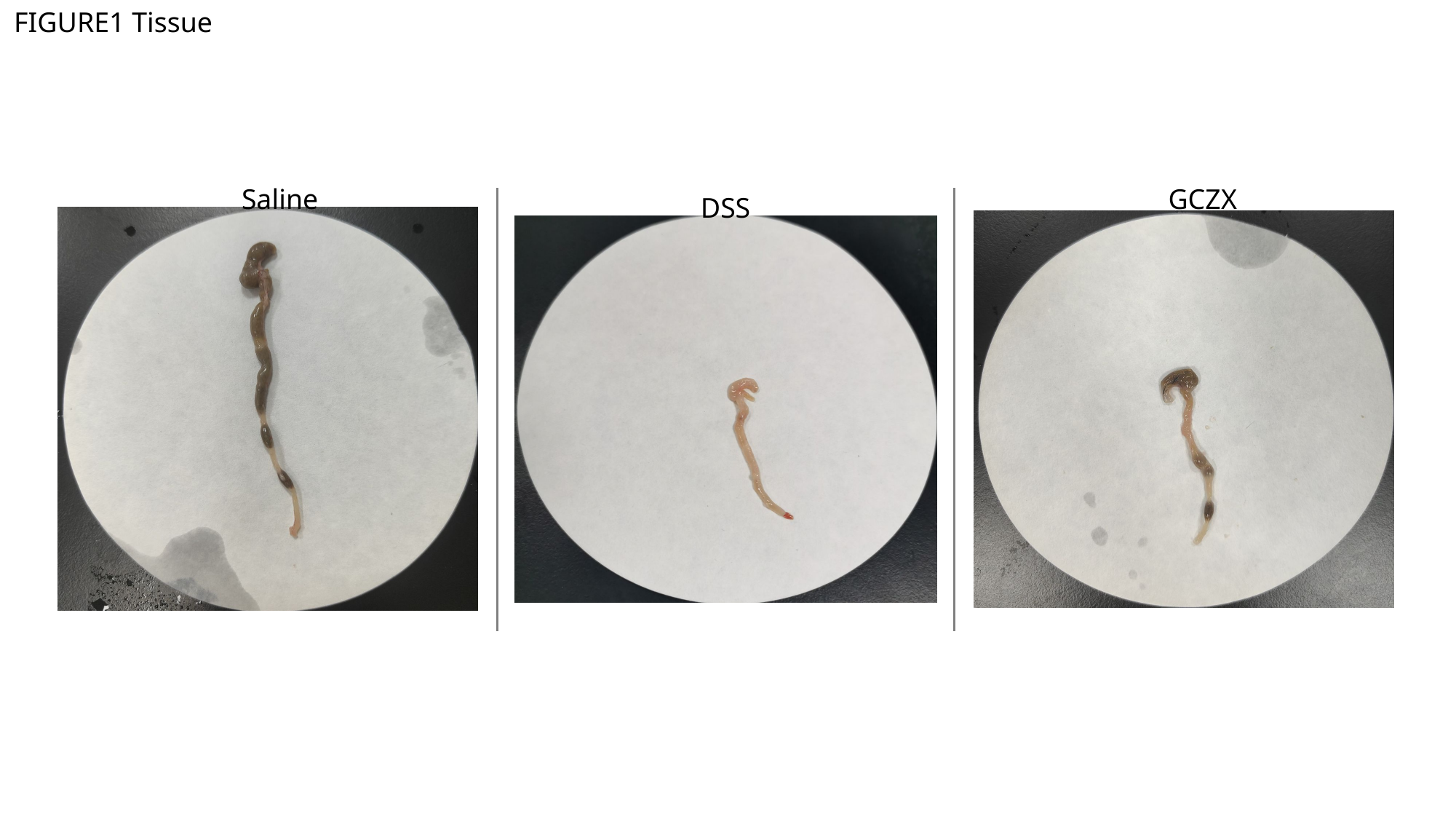

FIGURE1 Tissue
Saline
GCZX
DSS

## Slide 3
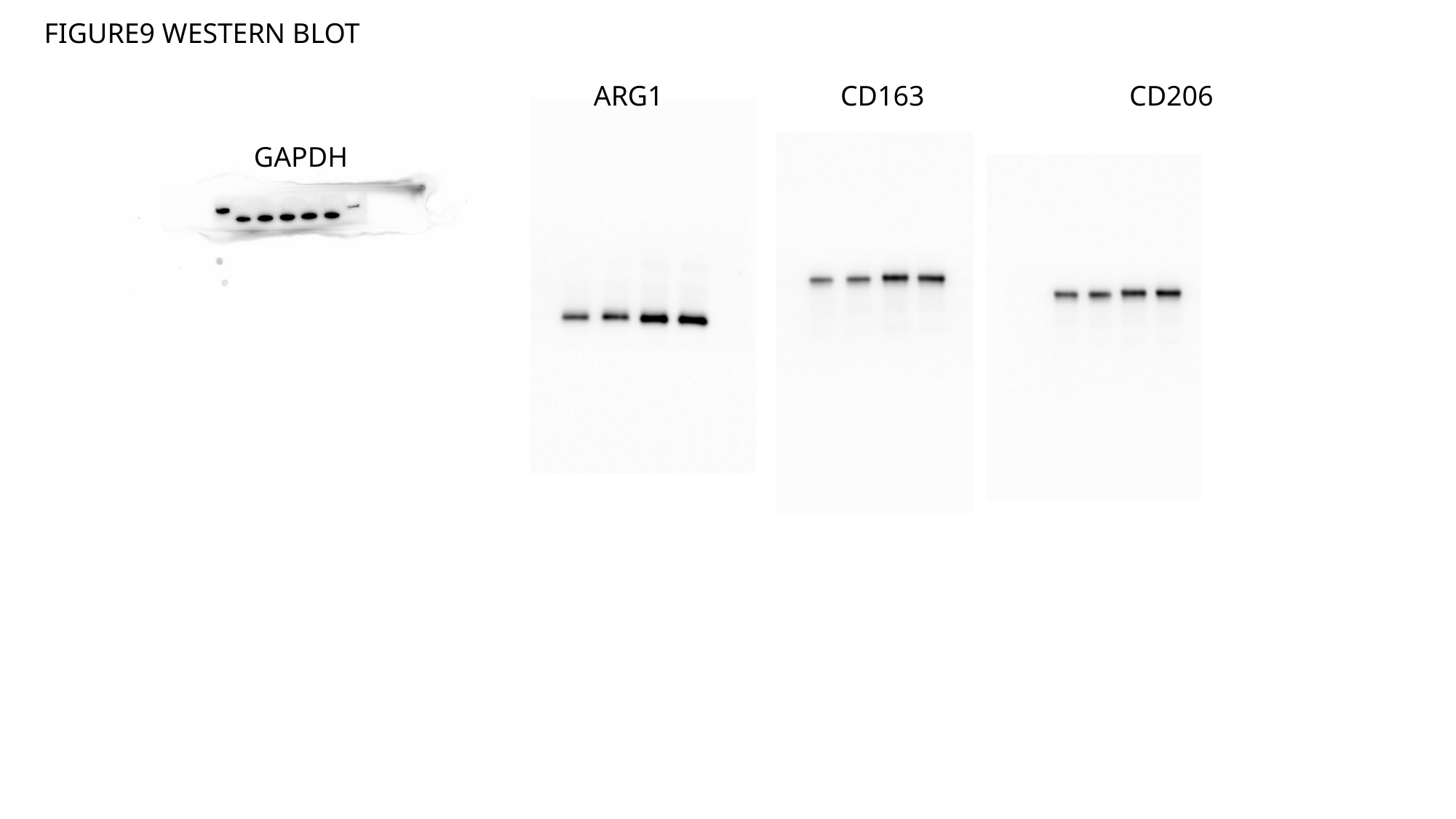

FIGURE9 WESTERN BLOT
ARG1
CD206
CD163
GAPDH

## Slide 4
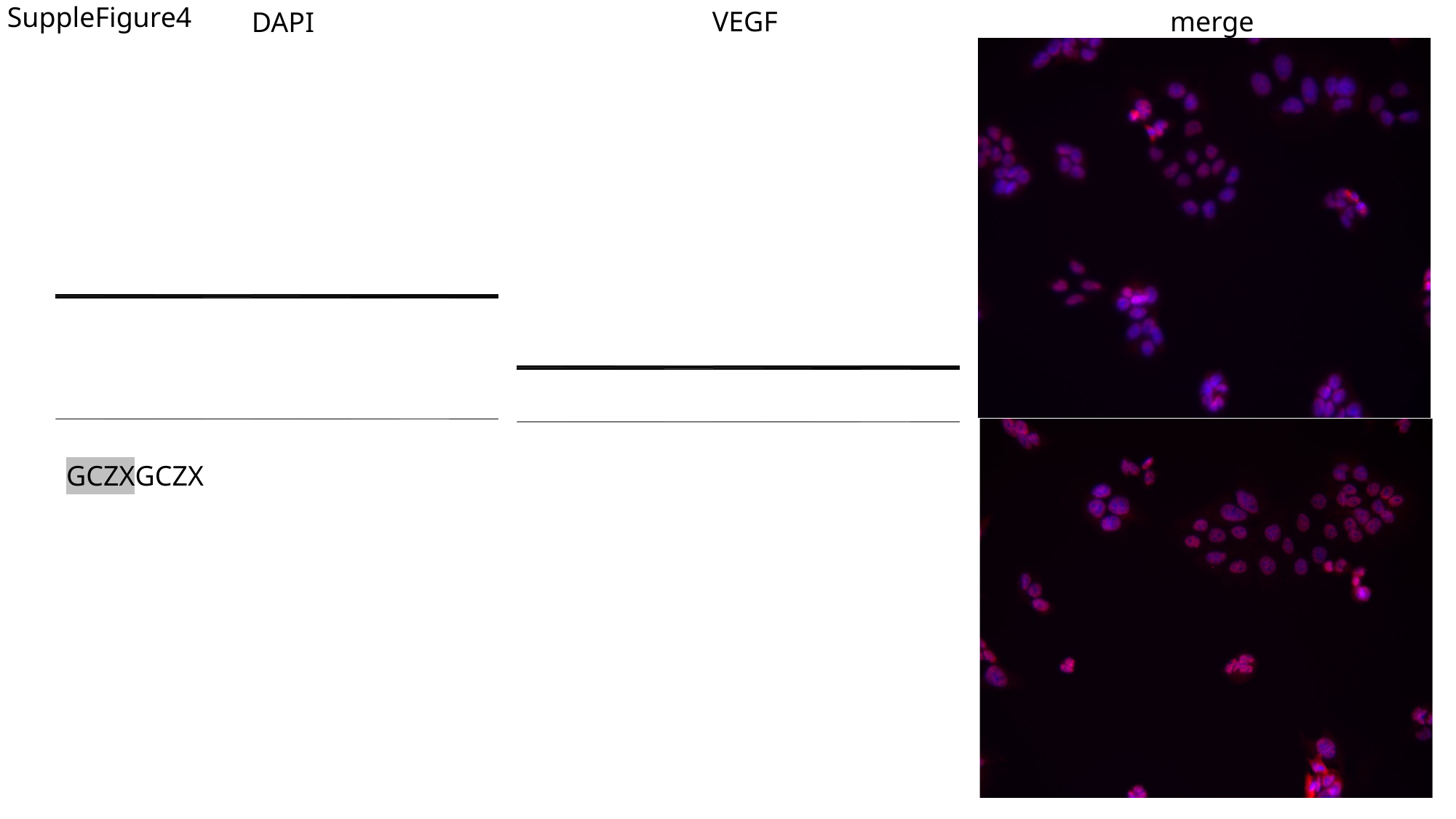

DAPI
SuppleFigure4
VEGF
merge
GCZXGCZX
