# Supplementary material for: The Pharmacological Mechanism of Guchangzhixie Capsule Against Experimental Colitis
Source: Front Pharmacol. 2021 Nov 18;12:762603. doi: 10.3389/fphar.2021.762603 (PMC8637769; doi:10.3389/fphar.2021.762603)
Supplement: Supplementary file 7 [file Table5.DOCX]

Supplementary method

Principal Coordinates Analysis (PCoA) is a dimension reduction sorting method, the principle is to assume that there are data that can measure the difference or distance among samples, then a rectangular coordinate system can be constructed. Samples are represented by dots, square of the euclidean distance between dots equals to the original difference data, so quantitative conversion of data can be realized, the major elements and structure can be extracted from multidimensional data. PERMANOVA analysis is known as Displacement multivariate analysis and was performed to test whether there is a significant difference between samples from different groups.

Similar to PCoA, Non-MetricMulti-Dimensional Scaling (NMDS) analysis is a sorting method suitable, it’s a data analysis method that can simplify the samples from multi-dimensional space to low-dimensional space for positioning, analysis and classification, while retaining the original relationship between objects. The difference between groups or within groups can be seen from the distribution of samples. The original design of NMDS is to overcome the shortcoming (linear model) of the previous sorting method including PCoA. PERMANOVA analysis was also performed.

Unweighted Pair-group Method with Arithmetic Mean (UPGMA) analysis is a commonly used clustering analysis method for sample hierarchical clustering. The principle is to assume that number of divergences occur in each lineage is the same, which means the replacement rate of nucleotides or amino acids is equal and constant. After each divergence, the length of branches from the common ancestor node to the two OTUs is the same.

Sample heatmap analysis Heatmap can get the distance matrix between samples by using distance algorithms (binary), the difference between samples can be visually seen according to the change of color gradient.

LEfSe (Linear discriminant analysis Effect Size) is an algorithm for High-Dimensional biomarker discovery and explanation that identifies genomic features (genes, pathways, or taxa) characterizing the differences between two or more biological conditions[1]. It emphasizes both statistical significance and biological relevance, allowing researchers to identify differentially abundant features that are also consistent with biologically meaningful categories. We used the non-parametric factorial Kruskal-Wallis (KW) sum-rank test to detect features with significant differential abundance with respect to the class; biological significance is subsequently investigated using a set of pairwise tests among subclasses using the (unpaired) Wilcoxon rank-sum test. LEfSe using Linear Discriminant Analysis was performed to estimate the effect size of each differentially abundant feature.

Reference

[1] N. Segata, J. Izard, L. Waldron, D. Gevers, L. Miropolsky, W.S. Garrett, and C. Huttenhower, Metagenomic biomarker discovery and explanation. Genome Biol 12 (2011) R60.
